# Targeting GPX4 to Induce Ferroptosis Overcomes Chemoresistance Mediated by the *PAX8‐AS1*/GPX4 Axis in Intrahepatic Cholangiocarcinoma

**DOI:** 10.1002/advs.202501042

**Published:** 2025-05-20

**Authors:** Zhi‐Wen Chen, Ji‐Jun Shan, Mo Chen, Zong Wu, Yi‐Ming Zhao, Hong‐Xu Zhu, Xin Jin, Yi‐Xiu Wang, Yi‐Bin Wu, Zhen Xiang, Zhi‐Wen Ding, Zhen‐Hai Lin, Long‐Rong Wang, Lu Wang

**Affiliations:** ^1^ Department of Hepatic Surgery Fudan University Shanghai Cancer Center Shanghai Medical College Fudan University Shanghai 200032 China

**Keywords:** chemoresistance, ferroptosis, GPX4, intrahepatic cholangiocarcinoma, PAX8‐AS1

## Abstract

The standard regimen of gemcitabine combined with cisplatin offers limited clinical benefits in the treatment of advanced intrahepatic cholangiocarcinoma (ICC) due to intrinsic or acquired resistance. Currently, effective biomarkers to predict and improve chemotherapy resistance in ICC are lacking. Here, it is reported that a long non‐coding RNA (lncRNA), *PAX8‐AS1*, reduces the efficacy of standard chemotherapeutic drugs. Mechanistically, *PAX8‐AS1* activates NRF2 by binding to p62, thereby promoting GPX4 transcription, and stabilizes *GPX4* mRNA through interaction with IGF2BP3. The *PAX8‐AS1*/GPX4 axis inhibits ferroptosis and promotes resistance to gemcitabine and cisplatin. In preclinical models, the combination of the GPX4 inhibitor JKE‐1674 with gemcitabine and cisplatin exhibits superior antitumor efficacy. These findings suggest a promising therapeutic strategy to improve chemotherapy efficacy in advanced ICC.

## Graphical abstract

*PAX8‐AS1* drives chemoresistance in intrahepatic cholangiocarcinoma by activating NRF2‐mediated GPX4 transcription and stabilizing *GPX4* mRNA via IGF2BP3. Targeting the *PAX8‐AS1*/GPX4 axis with a GPX4 inhibitor enhances the efficacy of gemcitabine and cisplatin in preclinical models, offering a promising strategy to overcome chemotherapy resistance in advanced intrahepatic cholangiocarcinoma.

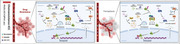

## 1 Introduction

Intrahepatic cholangiocarcinoma (ICC), the second most common primary liver malignancy, poses a significant therapeutic challenge due to its rising global incidence and persistently dismal prognosis.^[^ ^1^ ^]^ Although radical surgical resection offering the only potential cure, fewer than 40% of patients are eligible at the time of diagnosis.^[^ ^2^ ^]^ For the majority with advanced disease, systemic chemotherapy remains the cornerstone of treatment; however, the standard gemcitabine‐cisplatin regimen modestly prolongs survival, yielding a median overall survival of less than one year.^[^ ^3^ ^]^ This therapeutic stagnation highlights an urgent need to elucidate the molecular mechanisms driving chemotherapy resistance, which remains poorly understood.

Emerging evidence implicates ferroptosis, an iron‐dependent form of regulated cell death driven by lipid peroxidation, as a critical determinant of therapeutic response in various malignancies.^[^ ^4^ ^]^ Chemoresistant cells often evade ferroptosis through the action of antioxidant enzymes such as glutathione peroxidase 4 (GPX4), which detoxifies lipid peroxides. Despite its recognized importance, the role of ferroptosis in ICC chemotherapy resistance remains largely unexplored. Furthermore, although long non‐coding RNAs (lncRNAs) have emerged as key epigenetic regulators of tumor progression and drug resistance,^[^ ^5^ ^]^ their potential involvement in ferroptosis‐mediated chemoresistance in ICC has yet to be clarified. Addressing these gaps could unveil novel predictive biomarkers and therapeutic strategies to overcome treatment failure.

Here, we identify the lncRNA PAX8‐AS1 as a pivotal mediator of chemoresistance in ICC. Our study demonstrates that PAX8‐AS1 suppresses ferroptosis through a dual mechanism: 1) by binding p62 to enhance its interaction with KEAP1, thereby stabilizing NRF2 and transcriptionally upregulating GPX4, and 2) by interacting with IGF2BP3 to stabilize GPX4 mRNA. This coordinated increase of GPX4 expression confers robust resistance to chemotherapy‐induced ferroptosis. Crucially, combining the GPX4 inhibitor JKE‐1674 with standard chemotherapy overcomes resistance in patient‐derived organoid and xenograft models. Our findings not only establish PAX8‐AS1 as a biomarker for predicting chemoresistance but also validate the induction of ferroptosis as a precision strategy for enhancing chemosensitivity in ICC.

## 2 Results

### 2.1 *PAX8‐AS1* is Highly Expressed in Chemotherapy‐Resistant Intrahepatic Cholangiocarcinoma

**Figure 1:**
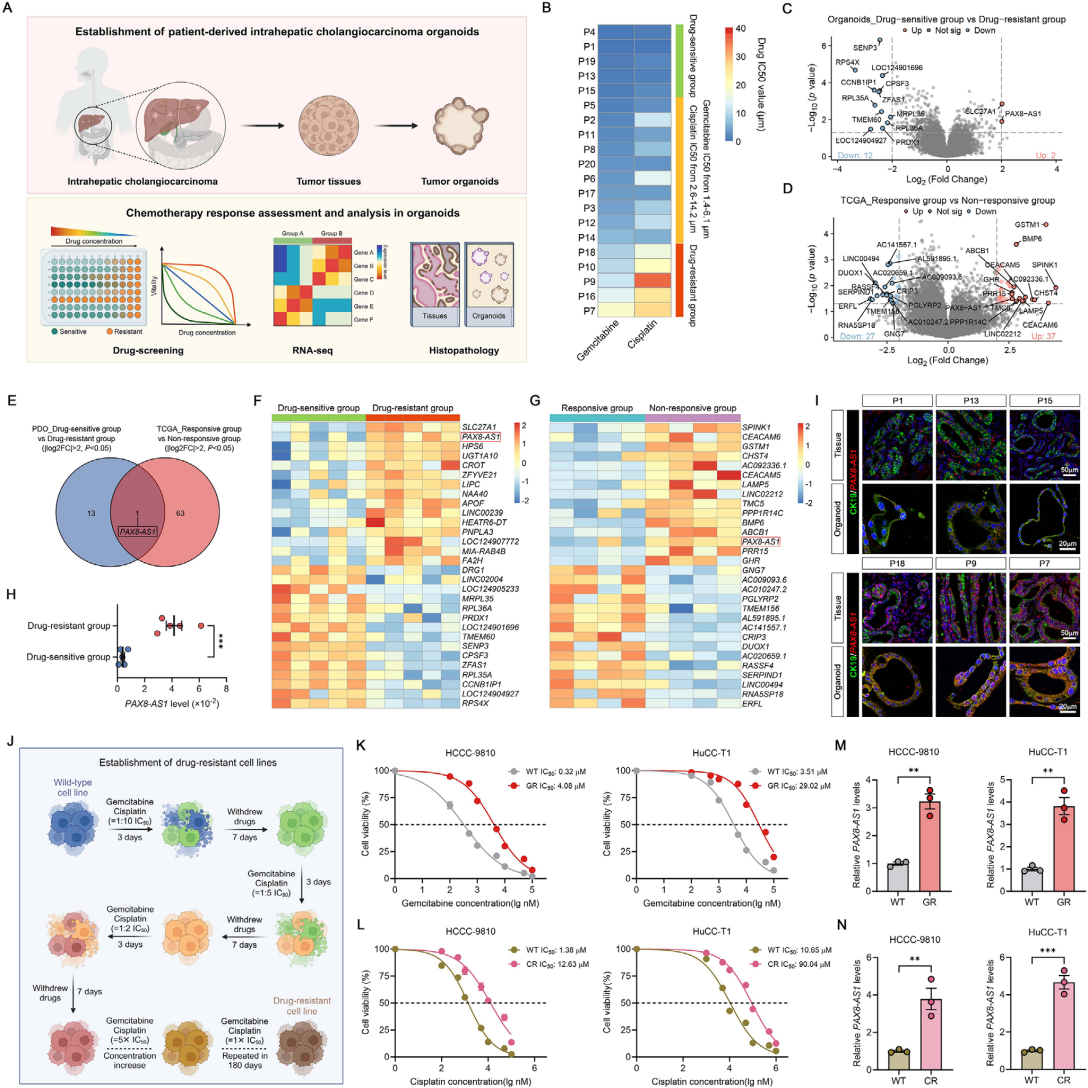
*PAX8‐AS1* is highly expressed in chemotherapy‐resistant intrahepatic cholangiocarcinoma. A) The schematic diagram illustrating the establishment and analysis of ICC PDOs, including drug screening, RNA sequencing, and histopathological analysis. B) Heatmap of IC_50_ values for gemcitabine and cisplatin in 20 ICC PDOs. C) Volcano plot of DEGs between drug‐sensitive and drug‐resistant ICC PDOs (|log2 fold change| > 2, *P* < 0.05). D) Volcano plot of DEGs between chemotherapy‐responsive and non‐responsive TCGA_CHOL patients (|log2 fold change| > 2, *P* < 0.05). E) The Venn diagram illustrates the overlap of DEGs between drug‐sensitive/resistant ICC PDOs and chemotherapy‐responsive/non‐responsive TCGA_CHOL patients. F) Heatmap showing the expression levels of the top 15 most significantly upregulated or downregulated DEGs in drug‐sensitive and drug‐resistant ICC PDOs. G) Heatmap showing the expression levels of the top 15 most significantly upregulated or downregulated DEGs in chemotherapy‐responsive and non‐responsive TCGA_CHOL patients. H) *PAX8‐AS1* levels in drug‐sensitive and drug‐resistant organoid‐derived tissues, determined by qRT‐PCR. I) *PAX8‐AS1* RNA‐FISH detection in organoids and their derived tissues. J) Schematic diagram of establishing gemcitabine‐resistant (GR) or cisplatin‐resistant (CR) ICC cell lines. K) Dose‐response curve for gemcitabine in wild‐type (WT) and GR ICC cells. L) Dose‐response curve for cisplatin in WT and CR ICC cells; *n*  =  3 biologically independent samples. M) Relative levels of *PAX8‐AS1* in WT and GR ICC cells, determined by qRT‐PCR; *n*  =  3 biologically independent samples. N) Relative levels of *PAX8‐AS1* in WT and CR ICC cells, determined by qRT‐PCR; *n*  =  3 biologically independent samples. Data are the mean ± SEM. ^**^ *p* < 0.01, ^***^ *p* < 0.001. *P* values were determined by unpaired two‐tailed Student's *t*‐tests (H, M, N).

To investigate the mechanisms of chemotherapy resistance in ICC, we established an ICC organoid biobank from 20 patients (**Figure** **1**A). Hematoxylin and eosin (H&E) staining of the organoids and their corresponding tissues demonstrated that the ICC organoids preserved the histopathological features of the parental tumors (Figure S1A, Supporting Information). Transcriptomic profiling (Figure S1B, Supporting Information) and whole exome sequencing (WES) analyses (Figure S1C,D, Supporting Information) revealed that the organoids retained the transcriptomic and genomic features of the parental tumors. We treated these patient‐derived organoids (PDOs) with gemcitabine and cisplatin and then assessed drug sensitivity. We found that PDOs P4, P1, P19, P13, and P15 were relatively sensitive to chemotherapy drugs (defined as the drug‐sensitive group), whereas PDOs P18, P10, P9, P16, and P7 were resistant (defined as the drug‐resistant group) (Figure 1B). Additionally, patients from the TCGA_CHOL dataset who had received chemotherapy were classified into a responsive group (n = 4, complete response or partial response) and a non‐responsive group (n = 4, stable disease or progressive disease) based on their clinical responses (Table S1, Supporting Information). Subsequent differential expression analysis identified genes with significant dysregulation between groups (thresholds: |log2 fold change| > 2, *P* < 0.05), as visualized in the volcano plots (Figure 1C,D). To identify key genes associated with chemoresistance, we performed an intersection analysis of differentially expressed genes (DEGs) from both cohorts. The venn diagram revealed *PAX8‐AS1* as the sole overlapping candidate (Figure 1E). The expression profiles of the top 15 significantly upregulated or downregulated genes, including *PAX8‐AS1*, were visualized in the heatmaps (Figure 1F,G). Notably, *PAX8‐AS1* was significantly upregulated in both the drug‐resistant PDO group and the TCGA_CHOL non‐response group.

We further validated these findings using qRT‐PCR, confirming that *PAX8‐AS1* was highly expressed in tissues derived from the PDO drug‐resistant group (Figure 1H). RNA‐FISH analysis of organoids and their corresponding tissues demonstrated significantly higher levels of *PAX8‐AS1* in the drug‐resistant group (P18, P9, and P7) compared to the drug‐sensitive group (P1, P13, and P15) (Figure 1I). Furthermore, we established gemcitabine‐resistant (GR) and cisplatin‐resistant (CR) ICC cell lines by stepwise dose escalation (Figure 1J). These resistant cell lines showed at least an eightfold increase in IC_50_ for gemcitabine or cisplatin compared to their corresponding wild‐type cells (Figures 1K,L). We found through qRT‐PCR analysis that the expression level of *PAX8‐AS1* in the acquired drug‐resistant cell lines was also higher than in the corresponding wild‐type cells (Figure 1M,N).

### 2.2 *PAX8‐AS1* Contributes to Chemotherapy Resistance in ICC

**Figure 2:**
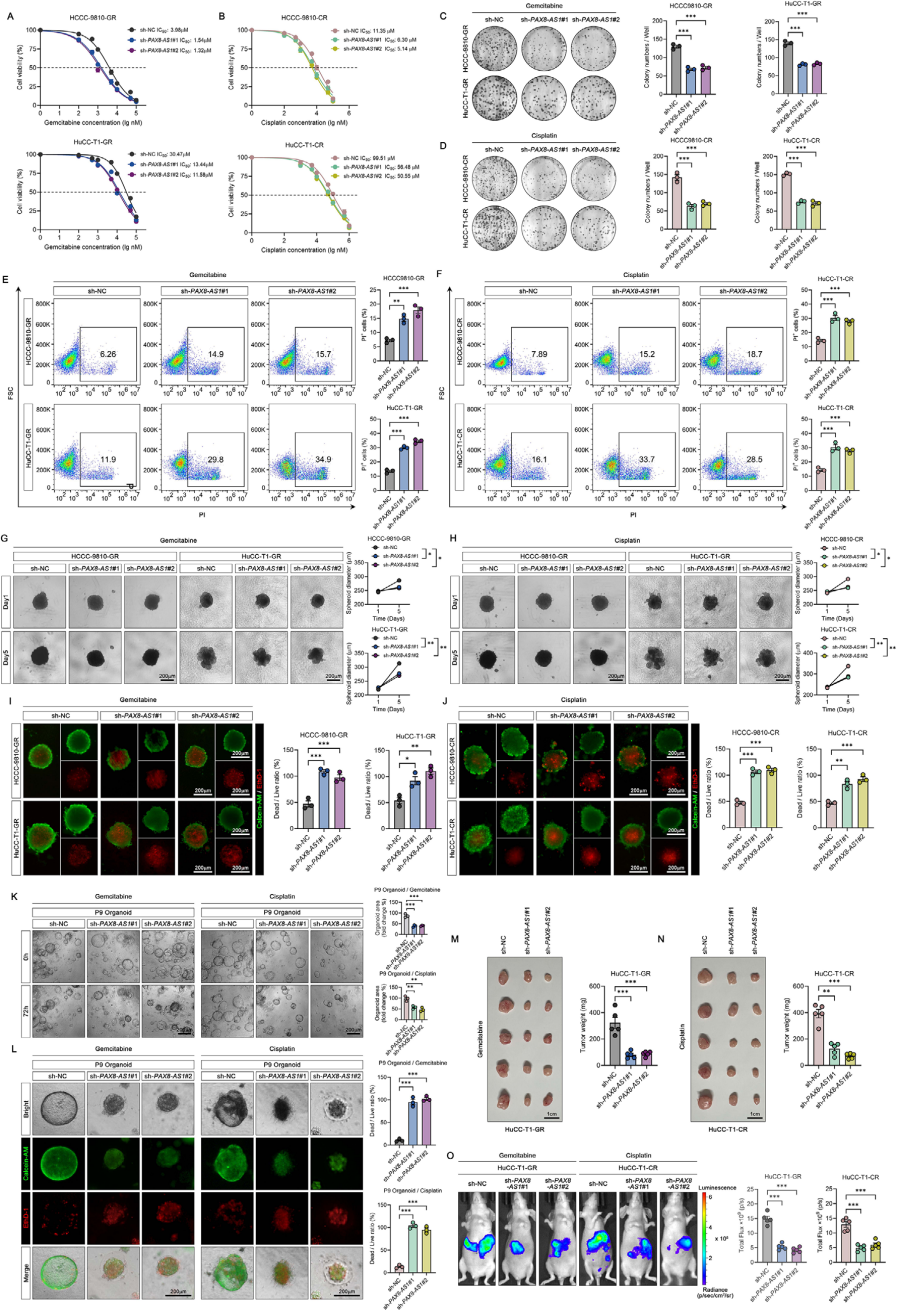
*PAX8‐AS1* contributes to drug resistance in vitro and in vivo. A,B) Dose‐response curves for gemcitabine and cisplatin in ICC cells with knockdown of *PAX8‐AS1*; *n*  =  3 biologically independent samples. C,D) Representative images and statistical analysis of colony formation in the indicated cells treated with gemcitabine or cisplatin at half the IC_50_ concentration for 72 h, followed by a two‐week incubation; *n*  =  3 biologically independent samples. E,F) Representative flow cytometry images and statistical analysis of PI‐stained cells after 72 h of treatment with gemcitabine or cisplatin at half the IC_50_ concentration; *n*  =  3 biologically independent samples. G,H) Representative images of ICC microtumor spheroid and quantification of their diameters on day one and day five after treatment with gemcitabine or cisplatin at half the IC_50_ concentration for 72 h; *n*  =  3 biologically independent samples. I,J) Representative images and fluorescence intensity ratios between dead (Eth‐D1) and live (Calcein‐AM) cells in ICC microtumor spheroids after 72 h of treatment with gemcitabine or cisplatin at half the IC_50_ concentration; *n*  =  3 biologically independent samples. K) Representative images and statistical analysis of PDOs treated with gemcitabine (5 µM) or cisplatin (10 µM) for 72 h; *n*  =  3 biologically independent samples. L) Representative images and fluorescence intensity ratios of dead (Eth‐D1) versus live (Calcein‐AM) cells in PDOs after 72 h of treatment with gemcitabine (5 µM) or cisplatin (10 µM); *n*  =  3 biologically independent samples. M,N) Images and weights of subcutaneous xenografts from the indicated ICC cells; *n*  =  5 mice per group. O) Representative images and statistical analysis of in vivo bioluminescence in the indicated orthotopic tumor models; *n*  =  5 mice per group. Data are the mean ± SEM. ^*^ *p* < 0.05, ^**^ *p* < 0.01, ^***^ *p* < 0.001. *P* values were determined by one‐way (C‐F, I‐O) or two‐way (G, H) ANOVA.

We conducted a series of drug sensitivity assays to determine the biological function of *PAX8‐AS1* in ICC. Results from cell viability and colony formation assays demonstrated that overexpression of *PAX8‐AS1* in wild‐type cells conferred resistance to gemcitabine or cisplatin, while knockdown of *PAX8‐AS1* sensitized drug‐resistant cells to these treatments (**Figure** **2**A–D; Figure S2A–D, Supporting Information). Propidium iodide (PI) staining (indicating cell death) revealed that under drug treatment, *PAX8‐AS1* overexpression reduced cell death in wild‐type cells, whereas knockdown of *PAX8‐AS1* increased cell death in resistant cells (Figure 2E, F; Figure S2E, F, Supporting Information). We further validated these findings using 3D microtumor spheroid models. Overexpression of *PAX8‐AS1* promoted spheroids growth under drug treatment and reduced the ratio of dead cells (Eth‐D1 positive) to live cells (Calcein‐AM positive), while the opposite effect was observed with *PAX8‐AS1* knockdown in resistant cells (Figure 2G–J; Figure S2G–J, Supporting Information). In addition, *PAX8‐AS1* overexpression enhanced the growth and survival of sensitive PDOs after gemcitabine or cisplatin treatment, whereas knockdown of *PAX8‐AS1* in resistant PDOs increased their drug sensitivity (Figure 2K, L; Figure S4A, B, Supporting Information).

To verify whether *PAX8‐AS1* contributes to drug resistance in ICC cells in vivo, we established mouse xenograft models using HuCC‐T1 and its drug‐resistant cells. In both subcutaneous tumor models and orthotopic models, overexpression of *PAX8‐AS1* in HuCC‐T1 cells enhanced resistance to gemcitabine and cisplatin. Conversely, the knockdown of *PAX8‐AS1* increased the sensitivity of HuCC‐T1‐GR and HuCC‐T1‐CR cells to these drugs (Figure 2M–O; Figure S4C–E, Supporting Information). Moreover, our results indicated that in the absence of drug treatment, *PAX8‐AS1* had no significant effect on the proliferation and death of ICC cells (both in vitro and in vivo) (Figures S3A–I and S5B–D, Supporting Information) and PDOs (Figure S5A, Supporting Information). These findings collectively demonstrate that *PAX8‐AS1* contributes to chemotherapy resistance in ICC.

### 2.3 *PAX8‐AS1* Regulates the KEAP1/NRF2 Pathway to Inhibit Ferroptosis

**Figure 3:**
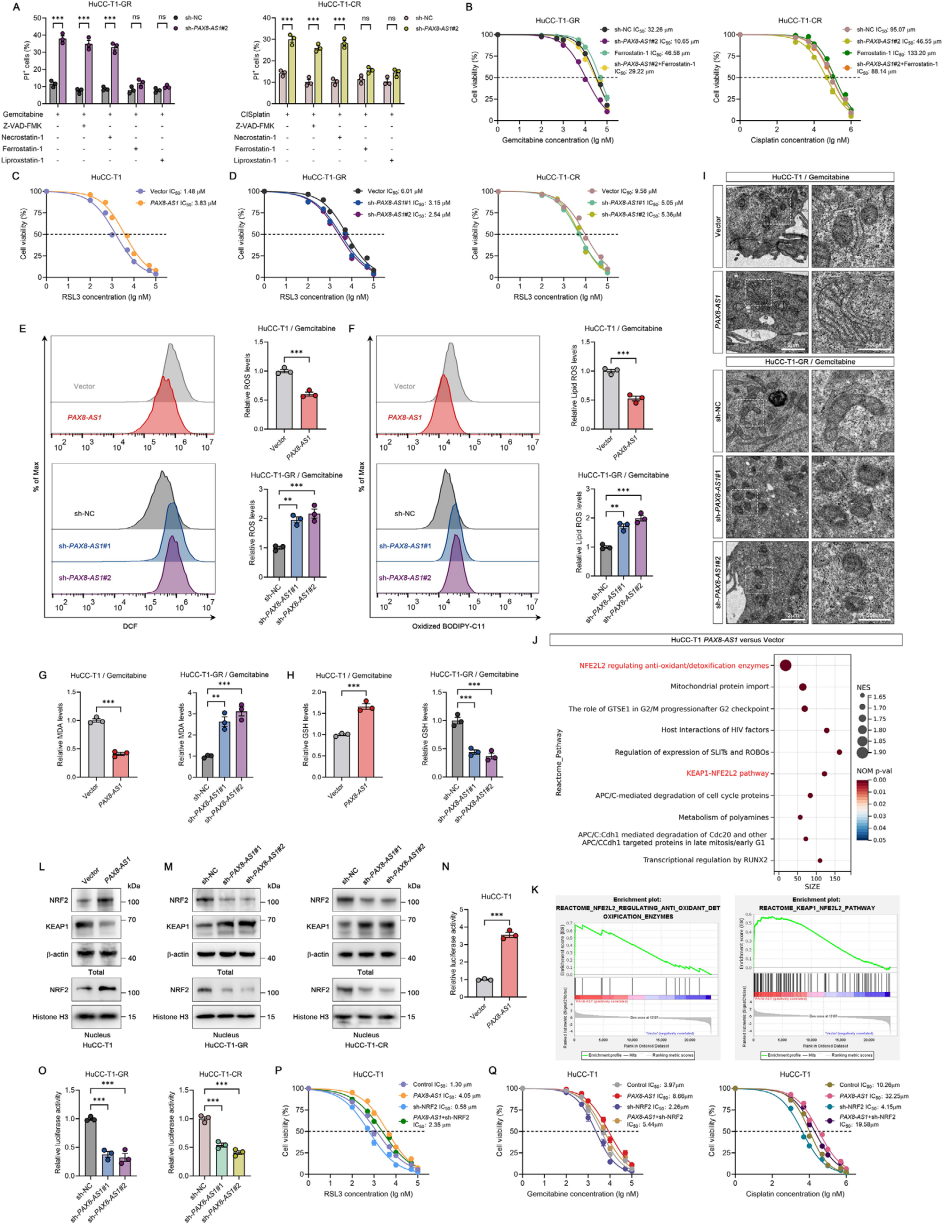
*PAX8‐AS1* regulates the Keap1/Nrf2 pathway to inhibit ferroptosis. A) Indicated cells were pretreated with DMSO, Z‐VAD‐FMK (20 µM, apoptosis inhibitor), necrostatin‐1 (10 µM, necroptosis inhibitor), ferrostatin‐1 (2 µM, ferroptosis inhibitor), or liproxstatin‐1 (0.5 µM, ferroptosis inhibitor) for 2 h, followed by co‐treatment with gemcitabine or cisplatin (at half the IC_50_ concentration) for 72 h. Cell death was measured by PI staining; *n*  =  3 biologically independent samples. B) Dose‐response curves for gemcitabine and cisplatin in the indicated cells. For ferrostatin‐1, cells were pretreated with 2 µM ferrostatin‐1 for 2 h, followed by co‐treatment with gemcitabine or cisplatin; *n*  =  3 biologically independent samples. C,D) Dose‐response curves for RSL3 in cells overexpressing or knocking down *PAX8‐AS1*; *n*  =  3 biologically independent samples. E) Representative flow cytometry images and statistical analysis of ROS levels in indicated cells after treatment with gemcitabine (at half the IC_50_ concentration) for 72 h; *n*  =  3 biologically independent samples. F) Representative flow cytometry images and statistical analysis of lipid peroxidation levels in indicated cells after treatment with gemcitabine (at half the IC_50_ concentration) for 72 h; *n*  =  3 biologically independent samples. G) Lipid oxidation product MDA levels in indicated cells after treatment with gemcitabine (at half the IC_50_ concentration) for 72 h; *n*  =  3 biologically independent samples. H) GSH levels in indicated cells after 72 h of treatment with gemcitabine (at half the IC_50_ concentration); *n*  =  3 biologically independent samples. I) Representative TEM images of mitochondrial morphology and ultrastructure in the indicated cells after 72 h of treatment with gemcitabine (at half the IC_50_ concentration); *n*  =  3 biologically independent samples. J,K) GSEA analysis revealed significantly enriched Reactome pathways in the *PAX8‐AS1* overexpression group. L,M) Western blot analysis of Nrf2 and Keap1 in cells overexpressing or knocking down *PAX8‐AS1*; *n*  =  3 biologically independent samples. N,O) pGL3‐ARE‐luc activity in cells overexpressing or knocking down *PAX8‐AS1*; *n*  =  3 biologically independent samples. P) Dose‐response curves for RSL3 in the indicated cells; *n*  =  3 biologically independent samples. Q) Dose‐response curves for gemcitabine and cisplatin in the indicated cells; *n*  =  3 biologically independent samples. Data are the mean ± SEM. ^*^ *p* < 0.05, ^**^ *p* < 0.01, ^***^ *p* < 0.001; ns, not significant. *P* values were determined by unpaired two‐tailed Student's *t*‐tests (E [upper], F [upper], G [left], H [left], N) and one‐way (E [lower], F [lower], G [right], H [right], O) or two‐way (A) ANOVA.

Next, we sought to understand how *PAX8‐AS1* contributes to chemotherapy resistance. It has been reported that chemotherapy drugs such as gemcitabine and cisplatin can induce apoptosis, necroptosis, and ferroptosis.^[^ ^6^ ^]^ To elucidate the mechanisms of cell death, we employed specific inhibitors targeting apoptosis, necroptosis, and ferroptosis. As previously reported, each inhibitor had varying degrees of inhibitory effects on cell death induced by gemcitabine or cisplatin alone. However, we found that in *PAX8‐AS1* knockdown cells, the cytotoxic effects of gemcitabine or cisplatin were significantly blocked by ferroptosis inhibitors (ferrostatin‐1 and liproxstatin‐1) (**Figure** **3**A). Furthermore, ferroptosis inhibitors restored the IC_50_ of gemcitabine or cisplatin in *PAX8‐AS1* knockdown cells (Figure 3B), suggesting that inhibition of ferroptosis may be an important pathway by which *PAX8‐AS1* promotes chemotherapy resistance.

To further validate this hypothesis, we examined the effect of *PAX8‐AS1* on sensitivity to the ferroptosis inducer RSL3. The results showed that overexpression of *PAX8‐AS1* increased the IC_50_ of RSL3 in HuCC‐T1 cells (Figure 3C), while knockdown of *PAX8‐AS1* sensitized resistant cells to RSL3 (Figure 3D). Additionally, we analyzed reactive oxygen species (ROS), lipid peroxidation, malondialdehyde (MDA), and glutathione (GSH) levels, and observed mitochondrial changes using transmission electron microscopy (TEM) to assess the level of ferroptosis. The results indicated that after treatment with gemcitabine and cisplatin, ROS, lipid peroxidation, and the lipid peroxidation product MDA levels decreased, GSH levels increased, and mitochondrial ultrastructure recovered in the *PAX8‐AS1* overexpression group, suggesting a reduced level of ferroptosis. In contrast, ferroptosis levels increased in resistant cells after *PAX8‐AS1* knockdown (Figure 3E–I; Figure S6A–E, Supporting Information). These data suggest that *PAX8‐AS1* inhibits chemotherapy drug‐induced ferroptosis.

To explore the molecular mechanism by which *PAX8‐AS1* regulates ferroptosis, we performed transcriptome sequencing after overexpressing *PAX8‐AS1*. Gene set enrichment analysis (GSEA) results showed that NRF2 regulating antioxidant/detoxification enzymes and the KEAP1/NRF2 pathway were significantly enriched in the *PAX8‐AS1* overexpression group (Figure 3J,K). Furthermore, we divided the TCGA_CHOL dataset into a high *PAX8‐AS1* expression group (top 50%) and a low *PAX8‐AS1* expression group (bottom 50%) based on *PAX8‐AS1* expression levels. Interestingly, GSEA analysis indicated that the high *PAX8‐AS1* expression group was closely associated with nuclear events mediated by NRF2, NRF2 regulating antioxidant/detoxification enzymes, and the KEAP1/NRF2 pathway (Figure S6F,G, Supporting Information). Nuclear factor erythroid 2‐related factor 2 (NRF2), a critical regulator of redox homeostasis, plays a key role in the regulation of ferroptosis.^[^ ^7^ ^]^

To determine whether *PAX8‐AS1* regulates NRF2 signaling, we examined NRF2 and KEAP1 protein levels after modulating *PAX8‐AS1* expression. *PAX8‐AS1* overexpression increased NRF2 protein levels and decreased KEAP1 protein levels, while *PAX8‐AS1* knockdown had the opposite effects (Figure 3L,M). However, *PAX8‐AS1* had no significant effect on the mRNA levels of *NRF2* and *KEAP1* (Figure S6H–K, Supporting Information), suggesting that *PAX8‐AS1* regulates NRF2 expression post‐transcriptionally. Additionally, *PAX8‐AS1* overexpression induced NRF2 nuclear accumulation, which was reduced following *PAX8‐AS1* knockdown. (Figure 3L,M). As a transcription factor, NRF2 functions by binding to the antioxidant‐responsive element (ARE) in the promoters of downstream genes.^[^ ^8^ ^]^ Using luciferase reporter assays, we found that ARE‐luc activity was significantly increased by *PAX8‐AS1* overexpression and decreased by *PAX8‐AS1* knockdown (Figure 3N,O). Rescue assays further demonstrated that NRF2 knockdown substantially attenuated *PAX8‐AS1*‐mediated resistance to RSL3 and chemotherapy drugs (Figure 3P,Q). These results suggest that *PAX8‐AS1* likely modulates NRF2 activity to inhibit ferroptosis and promote chemotherapy resistance.

### 2.4 NRF2 Accumulation is Attributed to *PAX8‐AS1* Binding to p62

**Figure 4:**
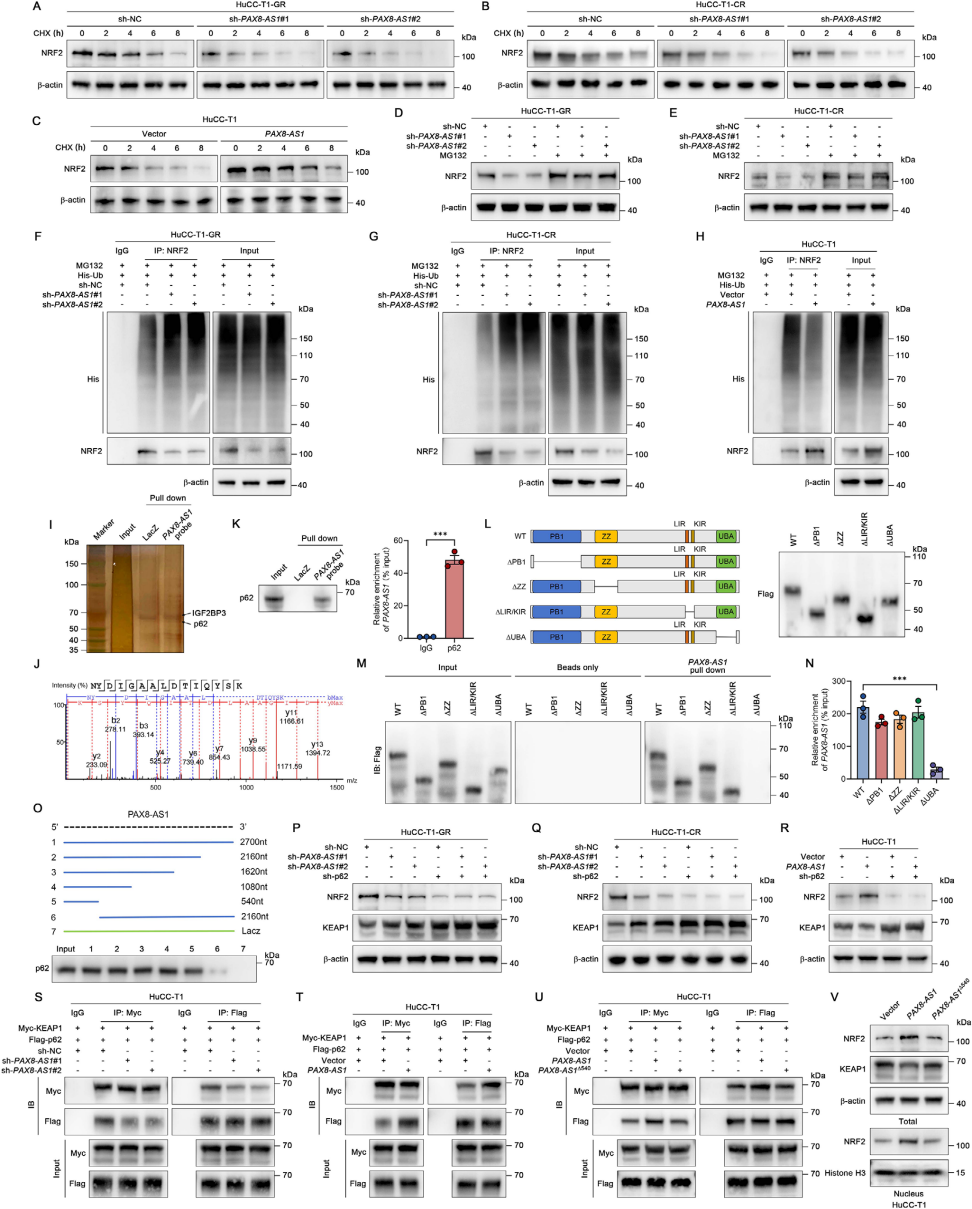
Nrf2 accumulation is attributed to *PAX8‐AS1* binding to p62. A–C) After treating cells with the protein synthesis inhibitor CHX (10 µg mL^−1^), Nrf2 protein degradation at different time points was detected by western blot assays; *n*  =  3 biologically independent samples. D,E) After treating *PAX8‐AS1* knockdown cells with the proteasome inhibitor MG132 (10 µM) for 8 h, Nrf2 protein levels were detected by western blot assays; *n*  =  3 biologically independent samples. F–H) Ub‐Nrf2 levels were determined by immunoprecipitation of Nrf2, followed by a western blot assay with an anti‐ubiquitin antibody in *PAX8‐AS1* knockdown or overexpressed cells; *n*  =  3 biologically independent samples. I,J) RNA pull‐down combined with mass spectrometry analysis revealed the interaction between *PAX8‐AS1* and p62. K) RNA pull‐down combined with western blotting (left) and RIP assays (right) were performed to detect the interaction between *PAX8‐AS1* and p62; *n*  =  3 biologically independent samples. L) Construction of p62 domain deletion mutants; *n*  =  3 biologically independent samples. M) The interaction between *PAX8‐AS1* and p62 mutants was detected by RNA pull‐down assays; *n*  =  3 biologically independent samples. N) The binding between *PAX8‐AS1* and different p62 mutants was assessed by RIP assays; *n*  =  3 biologically independent samples. O) The schematic diagram of *PAX8‐AS1* truncations (top) and RNA pull‐down assays to examine their association with p62 (bottom); *n*  =  3 biologically independent samples. P–R) Western blot analysis of Keap1 and Nrf2 protein levels in indicated cells after p62 knockdown; *n*  =  3 biologically independent samples. S–U) The binding affinity between p62 and Keap1 after *PAX8‐AS1* knockdown or overexpression was evaluated using Co‐IP assays; *n*  =  3 biologically independent samples. V) Western blot analysis of Nrf2 and Keap1 in the indicated cells; *n*  =  3 biologically independent samples. Data are the mean ± SEM. ^***^ *p* < 0.001. *P* values were determined by unpaired two‐tailed Student's *t*‐tests (K [right]) and one‐way ANOVA (N).

We hypothesized that *PAX8‐AS1* might regulate the protein stability of NRF2. To test this hypothesis, cells were treated with cycloheximide (CHX), a protein synthesis inhibitor. We found that *PAX8‐AS1* overexpression inhibited NRF2 protein degradation, whereas *PAX8‐AS1* knockdown accelerated NRF2 degradation (**Figure** **4**A–C). Ubiquitination and subsequent proteasome‐mediated protein degradation are key mechanisms controlling intracellular NRF2 levels.^[^ ^9^ ^]^ Interestingly, the reduction of NRF2 induced by *PAX8‐AS1* knockdown was restored by MG132, a proteasome inhibitor (Figure 4D,E). Additionally, ubiquitinated NRF2 (Ub‐NRF2) increased in *PAX8‐AS1* knockdown cells, indicating that *PAX8‐AS1* knockdown promoted NRF2 ubiquitination (Figure 4F,G). Conversely, NRF2 ubiquitination decreased in cells overexpressing *PAX8‐AS1* (Figure 4H). These data suggest that *PAX8‐AS1* stabilizes NRF2 protein by inhibiting ubiquitin‐proteasome‐mediated degradation.

To identify the mediator by which *PAX8‐AS1* stabilizes NRF2 protein, we performed RNA pull‐down assays with biotin‐labeled *PAX8‐AS1* and LacZ (negative control), followed by mass spectrometry (MS) analysis. Notably, p62 was identified in the MS results (Figure 4I,J), and its interaction with PAX8‐AS1 was further confirmed by immunoblotting and RNA immunoprecipitation (RIP) assays (Figure 4K). As a classical autophagy receptor, p62 can bind KEAP1, reducing the binding affinity between NRF2 and KEAP1, and blocking NRF2 ubiquitination.^[^ ^10^ ^]^ We generated a series of p62 mutants to determine which domain of p62 contributes to its interaction with *PAX8‐AS1* (Figure 4L). *PAX8‐AS1* RNA pull‐down and RIP assays showed that deletion of the UBA domain of p62 significantly abolished its association with *PAX8‐AS1* (Figure 4M,N). Additionally, truncated *PAX8‐AS1* RNA pull‐down assays indicated that the 1–540 nt region of *PAX8‐AS1* is primarily responsible for interacting with p62 (Figure 4O).

To determine the critical role of p62 in the *PAX8‐AS1*‐regulated KEAP1/NRF2 pathway, we performed p62 knockdown in cells with either *PAX8‐AS1* knockdown or overexpression. We found that p62 knockdown reversed the effects of *PAX8‐AS1* on KEAP1 degradation and NRF2 stabilization (Figure 4P–R). Previous studies have established that p62 binds to KEAP1, facilitating its sequestration into autophagosomes for degradation, thereby preventing KEAP1‐mediated NRF2 degradation.^[^ ^10^ ^]^ Our investigations further demonstrated that *PAX8‐AS1*‐induced reduction in KEAP1 abundance was significantly blocked by the autophagy inhibitor chloroquine (CQ) but not by MG132 (Figure S7, Supporting Information). Additionally, p62 knockout abolished KEAP1 downregulation. These findings collectively indicate that *PAX8‐AS1* promotes autophagic degradation of KEAP1 through a p62‐dependent mechanism. To investigate whether PAX8‐AS1 modulates the binding affinity between p62 and KEAP1, we performed co‐immunoprecipitation (Co‐IP) assays. The results revealed that *PAX8‐AS1* knockdown markedly weakened the interaction between p62 and KEAP1, whereas *PAX8‐AS1* overexpression, but not *PAX8‐AS1* ^Δ540^ (lacking the 1–540 nt region), enhanced their binding (Figure 4S–U). Furthermore, compared with *PAX8‐AS1*, *PAX8‐AS1* ^Δ540^ failed to induce KEAP1 reduction, NRF2 stabilization, and nuclear accumulation (Figure 4V). In summary, *PAX8‐AS1* facilitates the interaction between p62 and KEAP1 by binding to p62, thereby stabilizing NRF2 and promoting its accumulation.

### 2.5 *PAX8‐AS1* Promotes p62 Phase Separation to Activate NRF2

**Figure 5:**
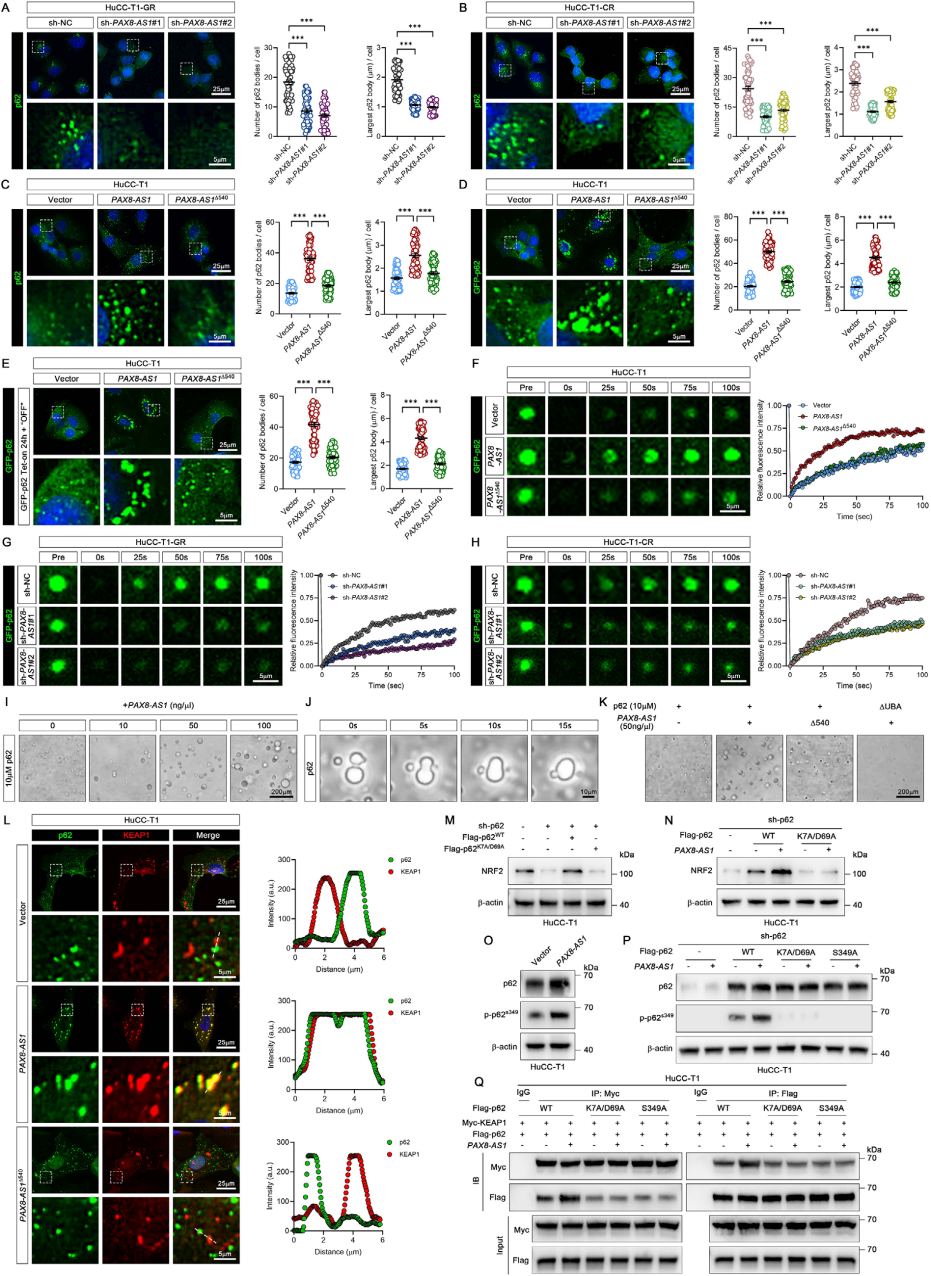
*PAX8‐AS1* promotes p62 phase separation to activate Nrf2. A,B) Representative immunofluorescence images of p62 protein in drug‐resistant HuCC‐T1 cells with *PAX8‐AS1* knockdown are shown. The number of p62 puncta > 0.5 µm per cell and their maximum diameter were quantified using ImageJ (*n* = 40 cells, from three independently plated wells). C) Representative immunofluorescence images of p62 protein in HuCC‐T1 cells overexpressing *PAX8‐AS1* or *PAX8‐AS1* ^Δ540^ are shown. The number of p62 puncta > 0.5 µm per cell and their maximum diameter were quantified using ImageJ (*n* = 40 cells, from three independently plated wells). D) Representative images of GFP‐p62 in HuCC‐T1 cells transfected with GFP‐p62 and overexpressing *PAX8‐AS1* or *PAX8‐AS1* ^Δ540^ are shown. The number of p62 puncta > 0.5 µm per cell and their maximum diameter were quantified using ImageJ (*n* = 40 cells, from three independently plated wells). E) HuCC‐T1 Tet‐on cells stably expressing GFP‐p62 were induced with doxycycline (Dox) for 24 h, followed by Dox removal to halt GFP‐p62 expression. Cells were then transfected with *PAX8‐AS1* or *PAX8‐AS1* ^Δ540^, and GFP‐p62 images were acquired 24 h later. The number of p62 puncta > 0.5 µm per cell and their maximum diameter were quantified using ImageJ (*n* = 40 cells, from three independently plated wells). F) FRAP analysis of GFP‐p62 puncta in HuCC‐T1 cells overexpressing either *PAX8‐AS1* or *PAX8‐AS1* ^Δ540^. (Left) Representative post‐bleach images at indicated time points. (Right) Fluorescence intensity recovery curves. (*n* = 9 puncta, from three independently plated wells). G,H) FRAP analysis of GFP‐p62 puncta in drug‐resistant HuCC‐T1 cells with *PAX8‐AS1* knockdown. (Left) Representative post‐bleach images at indicated time points. (Right) Fluorescence intensity recovery curves. (*n* = 9 puncta, from three independently plated wells). I) Representative phase separation images of purified p62 protein upon addition of varying concentrations of *PAX8‐AS1*; *n*  =  3 biologically independent samples. J) Representative fusion images of p62 protein droplets; *n*  =  3 biologically independent samples. K) Representative Phase separation images of purified p62 or p62^ΔUBA^ proteins in the indicated groups; *n*  =  3 biologically independent samples. L) Representative immunofluorescence images of p62 and Keap1 proteins in HuCC‐T1 cells overexpressing *PAX8‐AS1* or *PAX8‐AS1* ^Δ540^ are shown. The corresponding fluorescence intensity profiles along the indicated line are presented; *n*  =  3 biologically independent samples. M) Western blot analysis of Nrf2 in HuCC‐T1 cells with p62 knockdown followed by overexpression of p62^WT^ or p62^K7A/D69A^; *n*  =  3 biologically independent samples. N) Western blot analysis of Nrf2 in the indicated cells; *n*  =  3 biologically independent samples. O) Western blot analysis of p62 and phospho‐p62^Ser349^ in HuCC‐T1 cells overexpressing *PAX8‐AS1*; *n*  =  3 biologically independent samples. P) Western blot analysis of p62 and phospho‐p62^Ser349^ in the indicated cells; *n*  =  3 biologically independent samples. Q) The binding affinity between p62 and Keap1 in the indicated cells was detected by Co‐IP assays; *n*  =  3 biologically independent samples. Data are the mean ± SEM. ^***^ *p* < 0.001. *P* values were determined by one‐way ANOVA (A‐E).

A significant reduction in the number and size of p62 puncta was observed in resistant cells with *PAX8‐AS1* knockdown (**Figure** **5**A,B), whereas *PAX8‐AS1* overexpression in HuCC‐T1 cells enhanced p62 puncta formation (Figure 5C). Additionally, *PAX8‐AS1* ^Δ540^ showed minimal effects on p62 aggregation. (Figure 5C). Consistent with the results for endogenous p62, an increase in the number and size of p62 puncta was observed in HuCC‐T1 cells expressing GFP‐p62 after overexpression of *PAX8‐AS1* but not *PAX8‐AS1* ^Δ540^ (Figure 5D). The p62 protein exhibits viscous liquid‐like properties, enabling it to form p62 bodies via liquid‐liquid phase separation (LLPS).^[^ ^11^ ^]^ It has been reported that p62 bodies serve as platforms for NRF2 activation by sequestering KEAP1.^[^ ^12^ ^]^ We hypothesized that *PAX8‐AS1* promotes the formation of p62 bodies, thereby activating the KEAP1/NRF2 pathway. To exclude any potential impact of *PAX8‐AS1* on p62 expression that might affect p62 body formation, we generated Tet‐on inducible HuCC‐T1 cells stably expressing GFP‐p62. We first induced GFP‐p62 expression and then turned it off when *PAX8‐AS1* was transfected into the cells. Consistently, *PAX8‐AS1* promoted p62 body formation, but *PAX8‐AS1* ^Δ540^ had no significant effect (Figure 5E). Since p62 bodies possess fluidity, we examined whether *PAX8‐AS1* promotes p62 phase condensation using fluorescence recovery after photobleaching (FRAP) assays. GFP‐p62 puncta fluorescence recovered rapidly in the *PAX8‐AS1* overexpression group but showed limited recovery in the *PAX8‐AS1* ^Δ540^ overexpression group (Figure 5F). Moreover, *PAX8‐AS1* knockdown slowed the recovery rate of GFP‐p62 puncta fluorescence after photobleaching (Figure 5G,H).

To investigate the effect of *PAX8‐AS1* on p62 droplet formation in vitro, we in vitro transcribed *PAX8‐AS1* and incubated it with purified p62 protein. The isolated p62 exhibited relatively low basal activity in phase separation, consistent with previous reports.^[^ ^13^ ^]^ Notably, the formation of p62 droplets was enhanced in the presence of *PAX8‐AS1*, and p62 droplet formation was dose‐dependent on the amount of *PAX8‐AS1* added (Figure 5I). These p62 droplets exhibited fusion properties (Figure 5J), indicating their liquid‐like properties. Furthermore, this enhanced effect was abolished in the *PAX8‐AS1* ^Δ540^ or p62^ΔUBA^ groups (Figure 5K). These data indicate that *PAX8‐AS1* induces phase separation of p62, promoting the formation of p62 droplets. Next, immunofluorescence analysis revealed that KEAP1 extensively colocalized within p62 bodies after *PAX8‐AS1* overexpression, whereas *PAX8‐AS1* ^Δ540^ failed to promote p62‐KEAP1 colocalization (Figure 5L), supporting the experimental data that *PAX8‐AS1* enhances the interaction between p62 and KEAP1.

To verify that *PAX8‐AS1* activates NRF2 by promoting p62 phase separation, we generated a phase separation‐deficient p62 K7A/D69A mutant (p62^K7A/D69A^). Upon p62 knockdown followed by overexpression of either wild‐type p62 (p62^WT^) or the mutant p62^K7A/D69A^, we found that overexpression of p62^WT^ restored the NRF2 reduction caused by p62 knockdown, but p62^K7A/D69A^ did not (Figure 5M). Importantly, *PAX8‐AS1* overexpression increased NRF2 expression in the p62^WT^ group but not in the p62^K7A/D69A^ group (Figure 5N), indicating that *PAX8‐AS1* upregulates NRF2 in a p62 phase separation‐dependent manner. Previous studies have shown that phosphorylation of p62 at Ser349 increases its affinity for KEAP1 by 30‐fold, thereby promoting NRF2 activation.^[^ ^14^ ^]^ We found a significant increase in p62 Ser349 phosphorylation following *PAX8‐AS1* overexpression (Figure 5O). Total p62 levels also increased, which may be attributed to NRF2 activation, given that p62 is recognized as a target gene of NRF2.^[^ ^15^ ^]^ To investigate whether *PAX8‐AS1*‐induced p62 phosphorylation depends on phase separation, we overexpressed p62^WT^, p62^K7A/D69A^, and p62^S349A^ (a phosphorylation‐deficient mutant at Ser349) in p62‐deficient cells. Except for p62^S349A^, p62^K7A/D69A^ showed minimal phosphorylation at the Ser349 site. Moreover, *PAX8‐AS1* could only promote Ser349 phosphorylation of p62^WT^, not of p62^K7A/D69A^ or p62^S349A^ (Figure 5P). In addition, Co‐IP results demonstrated that *PAX8‐AS1* failed to enhance the interaction between KEAP1 and either p62^K7A/D69A^ or p62^S349A^ (Figure 5Q). These findings suggest that *PAX8‐AS1* promotes p62 phase separation, which leads to Ser349 phosphorylation, thereby mediating the activation of the KEAP1/NRF2 pathway.

### 2.6 The *PAX8‐AS1*/GPX4 Axis Promotes Chemotherapy Resistance

**Figure 6:**
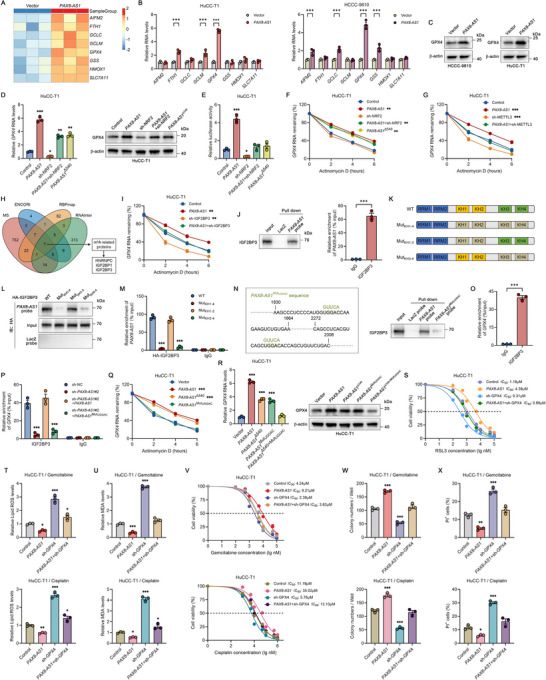
The *PAX8‐AS1*/GPX4 axis promotes chemotherapy resistance. A) A heatmap showing the mRNA expression profile of ferroptosis‐related genes in HuCC‐T1 cells overexpressing *PAX8‐AS1*. B) qRT‐PCR was used to detect mRNA levels of ferroptosis‐related genes in HuCC‐T1 and HCCC‐9810 cells overexpressing *PAX8‐AS1*; *n*  =  3 biologically independent samples. C) Western blot analysis of GPX4 in HuCC‐T1 and HCCC‐9810 cells overexpressing *PAX8‐AS1*; *n*  =  3 biologically independent samples. D) GPX4 mRNA and protein levels in the *PAX8‐AS1* overexpression group, *PAX8‐AS1* ^Δ540^ overexpression group, and *PAX8‐AS1* overexpression combined with Nrf2 knockdown group; *n*  =  3 biologically independent samples. E) Luciferase activity of the *GPX4* promoter in the indicated cells; *n*  =  3 biologically independent samples. F,G) qRT‐PCR analysis of *GPX4* mRNA levels in the indicated cells after treatment with Actinomycin D (2 µg mL^−1^) for different durations; *n*  =  3 biologically independent samples. H) Venn diagram illustrating candidate m6A‐related proteins that interact with *PAX8‐AS1*, identified by RNA pull‐down mass spectrometry (FDR > 1%, unique peptide > 1) and predicted by ENCORI (clipExpNum > 1), RBPmap (*p*‐value > 1%), and RNAInter (score > 0.5). I) qRT‐PCR analysis of *GPX4* mRNA levels in the indicated cells after treatment with Actinomycin D (2 µg mL^−1^) for different durations; *n*  =  3 biologically independent samples. J) RNA pull‐down combined with western blotting (left) and RIP assays (right) to investigate the interaction between *PAX8‐AS1* and IGF2BP3; *n*  =  3 biologically independent samples. K) Schematic diagram showing the RNA‐binding domain within the IGF2BP3 protein and a summary of IGF2BP3 variants. The gray box represents the KH domain after mutating GxxG to GEEG. L) RNA pull‐down assay showing the interaction between *PAX8‐AS1* and indicated IGF2BP3 variants; *n*  =  3 biologically independent samples. M) RIP assay showing the enrichment level of *PAX8‐AS1* in the indicated groups; *n*  =  3 biologically independent samples. N) The binding motif of IGF2BP3 in the *PAX8‐AS1* sequence is shown with a green background, while the mutated bases are indicated in green text (left). Levels of IGF2BP3 pulled down by *PAX8‐AS1* and *PAX8‐AS1* ^Mut^_UGGAC_ probes (right); *n*  =  3 biologically independent samples. O) RIP assay verifying the interaction between IGF2BP3 and *GPX4* mRNA; *n*  =  3 biologically independent samples. P) RIP assay showing the level of *GPX4* mRNA enriched by IGF2BP3 in the indicated groups; *n*  =  3 biologically independent samples. Q) qRT‐PCR analysis of *GPX4* mRNA levels in HuCC‐T1 cells overexpressing *PAX8‐AS1*, *PAX8‐AS1* ^Δ540^, or *PAX8‐AS1* ^Mut^_UGGAC_ after treatment with Actinomycin D (2 µg mL^−1^) for different durations; *n*  =  3 biologically independent samples. R) GPX4 mRNA and protein levels in HuCC‐T1 cells overexpressing *PAX8‐AS1*, *PAX8‐AS1* ^Δ540^, or *PAX8‐AS1* ^Mut^_UGGAC_; *n*  =  3 biologically independent samples. S) Dose‐response curves for RSL3 in the indicated cells; *n*  =  3 biologically independent samples. T) Lipid peroxidation levels in the indicated cells after treatment with gemcitabine or cisplatin at half the IC_50_ concentration for 72 h; *n*  =  3 biologically independent samples. U) Lipid oxidation product MDA levels in the indicated cells after treatment with gemcitabine or cisplatin at half the IC_50_ concentration for 72 h; *n*  =  3 biologically independent samples. V) Dose‐response curves for gemcitabine and cisplatin in the indicated cells; *n*  =  3 biologically independent samples. W) Colony numbers in the indicated cells treated with gemcitabine or cisplatin at half the IC_50_ concentration for 72 h, followed by a two‐week incubation; *n*  =  3 biologically independent samples. X) Flow cytometric analysis of PI‐positive cells in the indicated cells treated with half the IC_50_ concentration of gemcitabine or cisplatin for 72 h; *n*  =  3 biologically independent samples. Data are the mean ± SEM. ^*^ *p* < 0.05, ^**^ *p* < 0.01, ^***^ *p* < 0.001. *P* values were determined by unpaired two‐tailed Student's *t*‐tests (J [right], O) and one‐way (D [left], E, R, T, U, W, X) or two‐way (B, F, G, I, M, P, Q) ANOVA.

To better understand the molecular mechanism by which *PAX8‐AS1* inhibits ferroptosis, we analyzed the mRNA profile of ferroptosis‐related genes after overexpressing *PAX8‐AS1*. Several genes related to ferroptosis were upregulated in the *PAX8‐AS1* overexpression group, including *AIFM2*, *FTH1*, *GCLC*, *GCLM*, *GPX4*, *GSS*, *HMOX1*, and *SLC7A11* (**Figure** **6**A). To identify the downstream targets of *PAX8‐AS1*, we performed qRT‐PCR to assess mRNA levels of these genes and found a significant upregulation of *GPX4* mRNA in *PAX8‐AS1*‐overexpressing HuCC‐T1 and HCCC‐9810 cells (Figure 6B). Consistently, the protein levels of GPX4 were also increased in the *PAX8‐AS1* overexpression group (Figure 6C).

GPX4 utilizes GSH to reduce lipid hydroperoxides to their alcohol forms, which is a key step in preventing ferroptosis.^[^ ^16^ ^]^ Additionally, GPX4 is a known NRF2 target gene.^[^ ^7^, ^17^ ^]^ To verify whether *PAX8‐AS1* upregulates GPX4 by activating NRF2, we conducted rescue experiments. Unexpectedly, although NRF2 knockdown suppressed the *PAX8‐AS1*‐induced increase in GPX4 levels, GPX4 levels remained higher compared to the control group. Furthermore, *PAX8‐AS1* ^Δ540^ overexpression also significantly upregulated GPX4 levels (Figure 6D). These findings suggest that *PAX8‐AS1* may regulate GPX4 expression through the activation of NRF2 as well as other NRF2‐independent mechanisms. Considering that GPX4 mRNA and protein levels were elevated in both the *PAX8‐AS1* overexpression combined with NRF2 knockdown group and the *PAX8‐AS1* ^Δ540^ overexpression group, we deduced that an NRF2‐independent mechanism may regulate GPX4 expression at either the transcriptional or post‐transcriptional level. Since *GPX4* promoter luciferase activity in these groups was comparable to that of the control, post‐transcriptional regulation is likely the predominant mechanism (Figure 6E). As expected, we found that *GPX4* mRNA degradation was suppressed not only in the *PAX8‐AS1* overexpression group but also in the *PAX8‐AS1* overexpression combined with NRF2 knockdown group and the *PAX8‐AS1* ^Δ540^ overexpression group (Figure 6F).

Recent studies suggest that *N6*‐methyladenosine (m^6^A), the most abundant internal modification of mRNA, plays a critical role in regulating mRNA stability, and lncRNAs may influence this process.^[^ ^18^ ^]^ Although *PAX8‐AS1* did not alter overall m^6^A levels (Figure S8A, Supporting Information), knockdown of METTL3, a key m^6^A methyltransferase, reversed the *PAX8‐AS1*‐mediated stabilization of *GPX4* mRNA (Figure 6G). This indicates that *PAX8‐AS1* might stabilize *GPX4* mRNA via an m^6^A‐associated protein. By integrating computational predictions from online tools (ENCORI, RBPmap, and RNAInter) with mass spectrometry results from *PAX8‐AS1* RNA pull‐down assays (Table S2, Supporting Information), we identified seven overlapping candidates, three of which are m^6^A‐related (HNRNPC, IGF2BP1, and IGF2BP3) (Figure 6H). We then performed individual knockdowns of these three m6A‐related proteins to identify the mediator of *PAX8‐AS1*‐induced *GPX4* mRNA stabilization. We found that only the knockdown of IGF2BP3 impaired the *PAX8‐AS1*‐mediated stabilization of *GPX4* mRNA (Figure 6I), while knockdown of HNRNPC or IGF2BP1 had no significant effect (Figure S8B,C, Supporting Information). These findings suggest that *PAX8‐AS1* enhances *GPX4* mRNA stability through IGF2BP3. RNA pull‐down and RIP assays further confirmed the interaction between *PAX8‐AS1* and IGF2BP3 (Figures 4I and 6J). Previous studies have shown that the KH domains, particularly the GxxG motif, are crucial for interactions between IGF2BPs and RNA.^[^ ^19^ ^]^ We constructed multiple IGF2BP2 variants in which the GxxG motif was mutated to GEEG within the KH structural domain (Figure 6K). We found that the association between IGF2BP3 and *PAX8‐AS1* was barely detectable after double mutations in the KH3‐4 domains (Figure 6L,M). It has been reported that IGF2BP proteins preferentially bind the “UGGAC” motif,^[^ ^19b^ ^]^ which we identified within the *PAX8‐AS1* sequence (highlighted in green) (Figure 6N). Further, mutating “UGGAC” to “GUUCA” in *PAX8‐AS1* (*PAX8‐AS1* ^Mut^_UGGAC_) significantly weakened its interaction with IGF2BP3 in RNA pull‐down assays (Figure 6N). Our data suggest that IGF2BP3 binds *PAX8‐AS1* via its KH3 and KH4 domains, specifically recognizing the “UGGAC” motif.

*PAX8‐AS1* overexpression did not alter IGF2BP3 levels (Figure S8D, Supporting Information), indicating that *PAX8‐AS1* does not affect IGF2BP3 expression. Since IGF2BP3 can bind and stabilize *GPX4* mRNA,^[^ ^20^ ^]^ we wondered whether *PAX8‐AS1* plays a role in this process. RIP assays revealed that *GPX4* mRNA was indeed significantly enriched by IGF2BP3 (Figure 6O). Interestingly, *PAX8‐AS1* knockdown reduced the binding of IGF2BP3 to *GPX4* mRNA, which was restored by *PAX8‐AS1* overexpression but not by *PAX8‐AS1* ^Mut^_UGGAC_ overexpression (Figure 6P). Moreover, *PAX8‐AS1* ^Mut^_UGGAC_ failed to stabilize *GPX4* mRNA, indicating that *PAX8‐AS1* mediates *GPX4* mRNA stabilization through its interaction with IGF2BP3 (Figure 6Q). Next, we overexpressed a *PAX8‐AS1* plasmid with mutations in the IGF2BP3 and p62 binding site (*PAX8‐AS1* ^Δ540+Mut^_UGGAC_) in HuCC‐T1 cells and compared GPX4 levels with *PAX8‐AS1*, *PAX8‐AS1* ^Δ540^, or *PAX8‐AS1* ^Mut^_UGGAC_ overexpression groups. Overexpression of *PAX8‐AS1* ^Δ540^ or *PAX8‐AS1* ^Mut^_UGGAC_ partially upregulated GPX4 mRNA and protein levels, while GPX4 levels in *PAX8‐AS1* ^Δ540+Mut^_UGGAC_ overexpression cells were similar to those in the control group (Figure 6R). Based on the above data, we propose two mechanisms for *PAX8‐AS1*‐mediated GPX4 upregulation: promoting GPX4 transcription through NRF2 activation and enhancing *GPX4* mRNA stability via interaction with IGF2BP3.

Subsequently, a series of rescue experiments were conducted. We found that GPX4 knockdown reversed the *PAX8‐AS1*‐induced resistance to RSL3 (Figure 6S) as well as the suppression of lipid peroxidation and MDA levels caused by *PAX8‐AS1* under gemcitabine or cisplatin treatment (Figure 6T,U). Moreover, GPX4 knockdown sensitized HuCC‐T1 cells to gemcitabine or cisplatin, reversed the increased IC_50_ of these drugs in *PAX8‐AS1*‐overexpressing cells, and attenuated the *PAX8‐AS1*‐mediated promotion of cell survival and inhibition of cell death following chemotherapy drug treatment (Figure 6V–X). Collectively, these findings suggest that *PAX8‐AS1* promotes chemoresistance by upregulating GPX4 to inhibit ferroptosis.

### 2.7 JKE‐1674 sSensitizes Drug‐Resistant ICC to Chemotherapy

**Figure 7:**
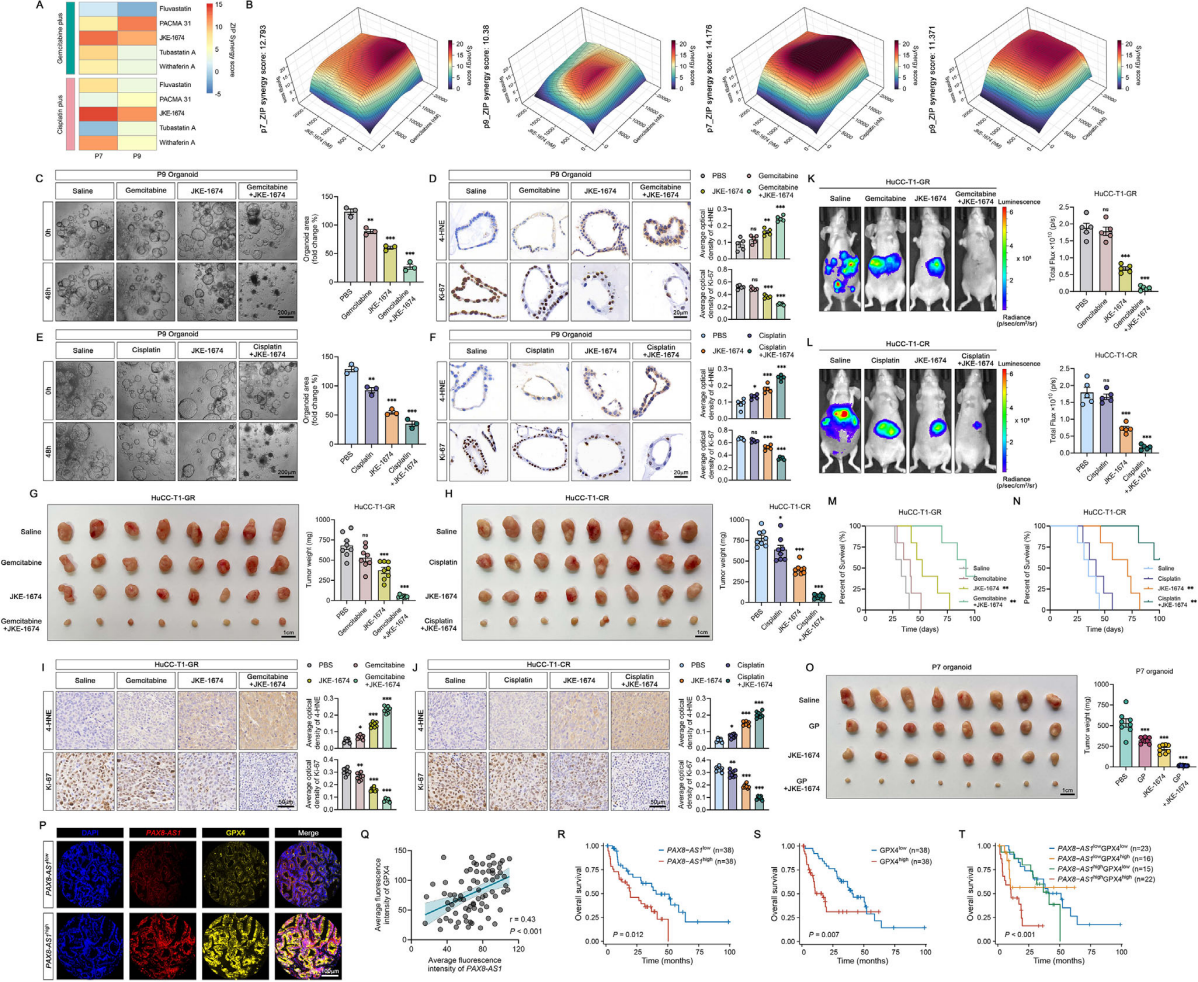
JKE‐1674 sensitizes drug‐resistant ICC to chemotherapy. A) A heatmap of synergy scores for GPX4 inhibitors (fluvastatin, PACMA 31, JKE‐1674, tubastatin A, and withaferin A) combined with gemcitabine or cisplatin in P7 and P9 organoids was analyzed with the SynergyFinder tool using the zero interaction potency (ZIP) model. B) 3D synergy maps of JKE‐1674 plus gemcitabine or cisplatin in PDOs after 72‐h treatment. C,E) Representative images and statistical analyses of PDOs treated with JKE‐1674 (1 µM) combined with gemcitabine (5 µM) or cisplatin (10 µM) for 72 h; *n*  =  3 biologically independent samples. D,F) Representative images and statistical analysis of Ki‐67 and 4‐HNE immunohistochemical staining in PDOs treated as indicated; *n*  =  3 biologically independent samples. G,H) Images and weights of subcutaneous xenografts derived from drug‐resistant HuCC‐T1 cells after the indicated treatments; *n*  =  8 mice per group. I,J) Representative images and statistical analysis of Ki‐67 and 4‐HNE immunohistochemistry in subcutaneous xenografts following the indicated treatments; *n*  =  8 mice per group. K,L) Representative images and statistical analysis of in vivo bioluminescence in the orthotopic tumor models with the indicated treatments; *n*  =  5 mice per group. M,N) Survival curves of orthotopic tumor‐bearing mice after the indicated treatments; *n*  =  5 mice per group. O) Images and weights of subcutaneous xenografts derived from P7 orgnoid following the indicated treatments; *n*  =  8 mice per group. P) Representative images of *PAX8‐AS1* FISH staining and GPX4 immunofluorescence staining in ICC tissue microarrays. Q) Pearson correlation analysis of fluorescence intensity between *PAX8‐AS1* FISH staining and GPX4 immunofluorescence staining in ICC tissue microarrays. R,S) Kaplan‐Meier survival analysis of *PAX8‐AS1* and GPX4 levels with overall survival (OS) in 76 ICC patients, using median levels as the cut‐off values. T) Kaplan‐Meier survival analysis of four subgroups (*PAX8‐AS1* ^low^/GPX4^low^, *PAX8‐AS1* ^low^/GPX4^high^, *PAX8‐AS1* ^high^/GPX4^low^ and *PAX8‐AS1* ^high^/GPX4^high^). Data are the mean ± SEM. ^*^ *p* < 0.05, ^**^ *p* < 0.01, ^***^ *p* < 0.001; ns, not significant. *P* values were determined by one‐way ANOVA (C‐L, O), Pearson correlation test (Q) and log‐rank test (M, N, R‐T).

Given that GPX4 inhibition can increase the sensitivity of ICC cells to chemotherapy drugs, we hypothesized that GPX4 inhibitors might be applicable for treating drug‐resistant ICC cells. However, the in vivo application of RSL3 is limited by its low solubility and poor pharmacokinetics.^[^ ^21^ ^]^ To address this, we selected five GPX4 inhibitors previously reported to be suitable for in vivo use (Fluvastatin, PACMA 31, JKE‐1674, Tubastatin A, and Withaferin A)^[^ ^22^ ^]^ to evaluate their synergistic effects with chemotherapy drugs in organoids. The results demonstrated that JKE‐1674 in combination with gemcitabine or cisplatin, exhibited a highly synergistic effect in P7 and P9 organoids (**Figure** **7**A,B). This combination treatment significantly inhibited organoid growth (Figure 7C,E), and immunohistochemistry (IHC) staining revealed lower levels of Ki‐67 and higher levels of 4‐hydroxynonenal (4‐HNE, a ferroptosis marker) in the organoids after the treatment (Figure 7D,F).

We then evaluated the therapeutic effect of combination treatment in vivo. In mice bearing HuCC‐T1‐GR or HuCC‐T1‐CR subcutaneous xenografts, the combination of JKE‐1674 with gemcitabine or cisplatin‐induced significant tumor regression (Figure 7G,H). IHC staining of the tumors revealed lower Ki‐67 levels and higher 4‐HNE levels (Figure 7I,J), with the results observed in organoids. Furthermore, JKE‐1674 alone or in combination with gemcitabine/cisplatin was well tolerated, as indicated by no significant changes in mouse body weight (Figure S9A, Supporting Information), absence of histopathological abnormalities in major organs such as the heart, liver, spleen, lungs, and kidneys (Figure S9B, Supporting Information), and normal blood biochemical parameters (ALT, AST, BUN, and CREA) (Figure S9C, Supporting Information). In orthotopic models implanted with HuCC‐T1‐GR or HuCC‐T1‐CR cells, the combination therapy demonstrated superior antitumor effects (Figure 7K,L) and improved survival rates (Figure 7M,N). Furthermore, we successfully established a patient‐derived organoid xenograft (PDOX) model using gemcitabine‐ and cisplatin‐resistant organoid (P7). The results demonstrated that JKE1674 significantly enhanced the therapeutic efficacy of the gemcitabine‐cisplatin (GP) combination regimen in the PDOX model (Figure 7O), highlighting its potential for translation.

We further investigated the clinical relevance of *PAX8‐AS1* and GPX4. According to the Gene Expression Profiling Interactive Analysis (GEPIA) database, high expression of *PAX8‐AS1* or GPX4 in TCGA_CHOL was associated with poor survival rates (Figure S9G,H, Supporting Information), and *PAX8‐AS1* levels were positively correlated with GPX4 levels (Figure S9I, Supporting Information). In a tissue microarray of 76 ICC tissues, we detected the levels of *PAX8‐AS1* and GPX4 and found that tissues with low *PAX8‐AS1* expression showed lower GPX4 levels, whereas high *PAX8‐AS1* levels were associated with increased GPX4 levels (Figure 7P). Furthermore, there was a positive correlation between the fluorescence intensities of *PAX8‐AS1* and GPX4 (Figure 7Q). Survival analysis indicated that patients with high expression of either *PAX8‐AS1* or GPX4 had shorter overall survival (OS) (Figure 7R,S). Notably, patients with high expression of both *PAX8‐AS1* and GPX4 exhibited the worst OS, which was significantly shorter compared to patients with low levels of both *PAX8‐AS1* and GPX4 (Figure 7T).

## 3 Discussion

**Figure 8:**
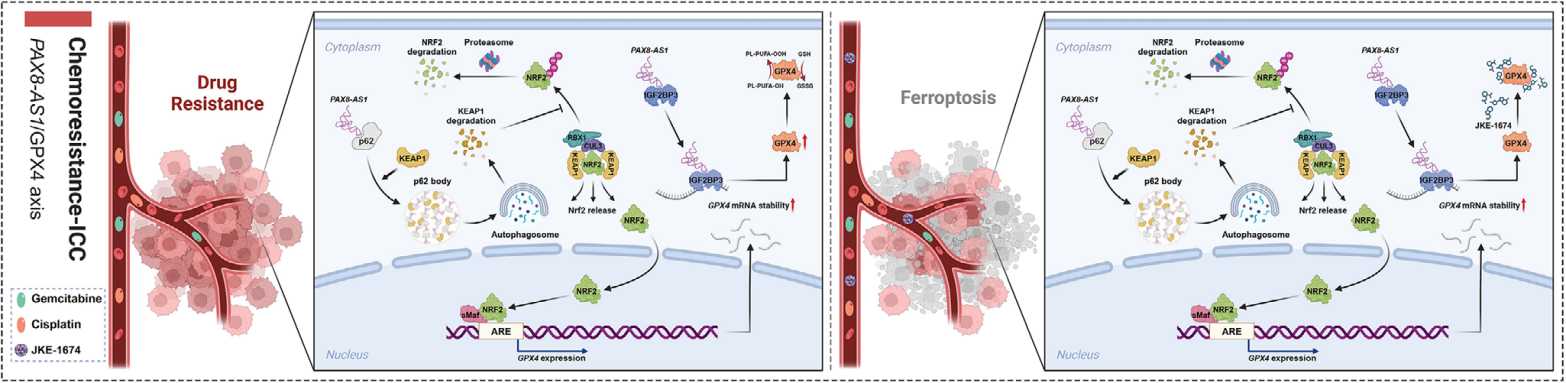
The GPX4 inhibitor JKE‐1674 overcomes chemoresistance mediated by the *PAX8‐AS1*/GPX4 axis in ICC. *PAX8‐AS1* interacts with p62 to promote the formation of p62 bodies, which sequester KEAP1 and subsequently activate NRF2, leading to increased transcription of GPX4. On the other hand, *PAX8‐AS1* also binds to IGF2BP3, enhancing the stability of *GPX4* mRNA. The upregulation of GPX4 inhibits chemotherapy‐induced ferroptosis. The application of the GPX4 inhibitor JKE‐1674 to counteract the *PAX8‐AS1*/GPX4 axis effectively improves the efficacy of chemotherapy.

Chemotherapy resistance is a major challenge for patients with advanced or unresectable ICC. The current standard chemotherapy regimen (gemcitabine combined with cisplatin) provides only marginal improvement in survival.^[^ ^3a^ ^]^ The complex mechanisms underlying chemotherapy resistance enable ICC cells to develop intrinsic and acquired resistance, which is a primary reason for poor drug responses.^[^ ^23^ ^]^ Therefore, it is urgent to explore the mechanisms driving chemotherapy resistance in ICC and to develop practical strategies to enhance the efficacy of gemcitabine and cisplatin, potentially broadening the therapeutic window for ICC patients. In this study, through transcriptomic analysis of PDOs and public datasets, we identified a lncRNA, *PAX8‐AS1*, which is abnormally overexpressed in both primary drug‐resistant PDOs and corresponding tissues, as well as in acquired drug‐resistant ICC cell lines. *PAX8‐AS1* interacts with p62 to promote the formation of p62 bodies and sequester KEAP1, thereby activating NRF2‐mediated transcription of GPX4. In addition, *PAX8‐AS1* binds to IGF2BP3 to enhance the stability of *GPX4* mRNA. The upregulation of GPX4 inhibits ferroptosis induced by gemcitabine and cisplatin, contributing to chemotherapy resistance in ICC cells. Using the GPX4 inhibitor JKE‐1674 to counteract the *PAX8‐AS1*/GPX4 axis effectively improved the efficacy of gemcitabine and cisplatin (Figure 8).

Currently, research on *PAX8‐AS1* is limited, mainly focusing on thyroid tumors and cervical cancer.^[^ ^24^ ^]^ Recently, Fang et al. reported that high expression of *PAX8‐AS1* is associated with poor prognosis and response to fluorouracil‐based chemotherapy in stage II colon cancer, suggesting its potential value in predicting chemotherapy efficacy in gastrointestinal tumors.^[^ ^25^ ^]^ In a study by Lu et al., *PAX8‐AS1* was significantly overexpressed in gemcitabine‐resistant cholangiocarcinoma cells but was not further investigated.^[^ ^26^ ^]^ Here, we are the first to focus on the role of *PAX8‐AS1* in chemotherapy resistance in ICC. High expression of *PAX8‐AS1* drives both intrinsic and acquired resistance in ICC cells and is associated with poor prognosis in ICC patients. Our findings provide novel insights into the poorly studied gene *PAX8‐AS1* and its function in gastrointestinal tumors. Importantly, we reveal its significant value in assessing chemotherapy efficacy for ICC.

In recent years, many studies have reported a close relationship between ferroptosis and tumor chemotherapy resistance.^[^ ^4^, ^27^ ^]^ Traditional chemotherapy drugs, such as gemcitabine and platinum compounds, can induce high levels of ROS, disrupting cellular redox homeostasis and subsequently triggering ferroptosis.^[^ ^6^, ^28^ ^]^ Studies have shown that modulating ferroptosis enables tumor cells to develop resistance to gemcitabine and platinum‐based drugs.^[^ ^29^ ^]^ In our study, we found that ferroptosis inhibitors significantly suppressed the death of chemoresistant cells with *PAX8‐AS1* knockdown under drug treatment, and that *PAX8‐AS1* reduce the level of ferroptosis in ICC cells. Subsequent transcriptomic analysis revealed that the NRF2 pathway was significantly enriched in the *PAX8‐AS1* high‐expression group. Notably, NRF2 is a key regulator of ferroptosis,^[^ ^7a^ ^]^ and activation of the NRF2 signaling pathway is a driving factor for both intrinsic and acquired chemotherapy resistance in tumors.^[^ ^7b^ ^]^ NRF2 is anchored in the cytoplasm by KEAP1, which serves as a substrate adaptor for the Cullin‐3‐dependent E3 ubiquitin ligase, promoting NRF2 ubiquitination and rapid proteasomal degradation.^[^ ^30^ ^]^ Meanwhile, p62 can compete with NRF2 for KEAP1 binding, sequestering KEAP1 into autophagosomes for degradation.^[^ ^10^ ^]^ Subsequently, NRF2 translocates to the nucleus and mediates the transcription of various protective antioxidant genes (including ferroptosis‐related genes), aiding cancer cells in resisting treatment.^[^ ^31^ ^]^

p62 is a classical autophagy adaptor protein that regulates various signaling pathways through interactions with multiple proteins.^[^ ^32^ ^]^ Although it lacks classical RNA‐binding domains, p62 has recently been identified as a novel RNA‐binding protein, capable of binding to various RNAs, including vtRNA, miRNA, and lncRNA.^[^ ^33^ ^]^ Here, we found that *PAX8‐AS1* binds to p62 and enhances its interaction with KEAP1, thereby activating NRF2. We further observed that the interaction between *PAX8‐AS1* and p62 promotes p62 phase separation to form p62 bodies. Previous studies have reported that phosphorylation of p62 at Ser349, located within the KIR (KEAP1‐interacting region), significantly enhances its binding to KEAP1 and mediates NRF2 activation. Moreover, Ser349 phosphorylation largely depends on the formation of p62 bodies.^[^ ^14^, ^34^ ^]^ Our data indicate that *PAX8‐AS1* facilitates the formation of p62 bodies, enhances Ser349 phosphorylation, and thereby activates NRF2. It remains unclear whether RNA binding to p62 directly influences its phase separation and the KEAP1/NRF2 pathway. Our study reveals the important role of the lncRNA *PAX8‐AS1* in promoting p62 phase condensation and subsequent NRF2 activation. However, the current evidence is insufficient to fully explain the mechanisms underlying this process, and further investigation is needed in the future.

We confirmed that GPX4 is the primary effector molecule through which *PAX8‐AS1* regulates ferroptosis. This finding aligns with expectations since GPX4 is a known target of NRF2.^[^ ^7^, ^17^ ^]^ However, further investigation revealed that the activation of NRF2 by *PAX8‐AS1* is not the only mechanism that upregulates GPX4. *PAX8‐AS1* also increases GPX4 levels through IGF2BP3. IGF2BP3 is a potent post‐transcriptional oncogene that stabilizes numerous target gene RNAs, promoting tumor growth, metastasis, and drug resistance.^[^ ^35^ ^]^ Previous studies have reported that IGF2BP3 promotes GPX4 expression to inhibit ferroptosis in glioma and lung adenocarcinoma.^[^ ^20^ ^]^ Whether this regulatory relationship plays a role in ICC and tumor chemotherapy resistance remains unknown. Additionally, although many studies have highlighted the importance of lncRNAs in mediating the stability of IGF2BP3 targets in cancer,^[^ ^36^ ^]^ it is unclear whether lncRNAs collaborate with IGF2BP3 to regulate ferroptosis‐related targets. Our data indicate that the interaction between *PAX8‐AS1* and IGF2BP3 facilitates the binding and stabilization of *GPX4* mRNA by IGF2BP3, thereby inhibiting ferroptosis and enabling ICC cells to resist chemotherapy drugs. Given the wide range of IGF2BP3 targets, further investigation is needed to determine whether the interaction between *PAX8‐AS1* and IGF2BP3 regulates the expression of other ferroptosis‐related genes or contributes to the selective binding of IGF2BP3 to *GPX4* mRNA.

GPX4 is a central regulator of ferroptosis, utilizing GSH as a substrate to convert lipid hydroperoxides into non‐toxic lipid alcohols (36). The survival of resistant persister cells depends on GPX4, and resistant cancer cells are highly sensitive to ferroptosis induced by GPX4 inhibition, making GPX4 an ideal target for overcoming tumor therapy resistance.^[^ ^37^ ^]^ Conventional GPX4 inhibitors (including RSL3, ML162, and ML210) are valuable tool compounds in cell culture studies, but their application in vivo is limited by low solubility and poor pharmacokinetics.^[^ ^21^ ^]^ In recent years, several studies have identified effective GPX4 inhibitors that are suitable for in vivo therapeutic applications.^[^ ^22^ ^]^ We tested the drug synergy of these emerging GPX4 inhibitors with chemotherapy drugs and evaluated the potential of GPX4 inhibition in vivo to overcome the chemoresistance of ICC cells. The results demonstrate that JKE‐1674 exhibits optimal synergy with gemcitabine and cisplatin. Furthermore, the combination therapy effectively suppressed the growth of chemotherapy‐resistant ICC cells across multiple preclinical models. As a structural analog of ML210, JKE‐1674 replaces the nitroisoxazole ring with an α‐nitroketoxime, enabling highly specific inhibition through covalent binding to the catalytic selenocysteine residue of GPX4.^[^ ^22a^ ^]^ This unique mechanism endows JKE‐1674 with potent ferroptosis‐inducing capacity, significantly amplifying chemotherapy‐induced oxidative damage. Additionally, JKE‐1674 demonstrates favorable in vivo stability, remaining detectable in mouse serum for up to 24 h after administration,^[^ ^22a^ ^]^ ensuring sustained pharmacological activity, which likely contributes to its superior performance. Both our findings and previous studies confirm that JKE‐1674 exhibits minimal toxicity in mice,^[^ ^22^, ^38^ ^]^ underscoring its favorable safety profile and highlighting its potential for clinical translation.

Our study has several limitations. First, although we characterized the role of the *PAX8‐AS1*/GPX4 axis in ICC, its generalizability to other cancer types remains unexplored. Validating its functional relevance across diverse malignancies could strengthen its broader therapeutic potential. Second, we did not establish a multidrug‐resistant (MDR) ICC cell line. While gemcitabine‐ and cisplatin‐resistant models were successfully generated to investigate mechanisms of chemoresistance, MDR models—characterized by simultaneous resistance to multiple structurally and mechanistically distinct agents—could provide a broader and more clinically relevant understanding of resistance. Third, given that ICC has entered the era of immunotherapy ^[^ ^39^ ^]^ and ferroptosis plays a multifaceted role in antitumor immunity,^[^ ^40^ ^]^ it remains unclear whether the *PAX8‐AS1*/GPX4 axis influences immune interactions or whether JKE‐1674‐induced ferroptosis can stimulate an antitumor immune response to overcome chemoresistance. Finally, although nanocarrier systems represent a promising strategy to improve the in vivo performance of hydrophobic GPX4 inhibitors, we did not evaluate such delivery approaches in the present study. In future work, we intend to explore nanoparticle‐based delivery systems for GPX4 inhibitors to broaden therapeutic options.

In summary, our study reveals the mechanism by which the lncRNA *PAX8‐AS1* promotes ICC chemotherapy resistance by upregulating GPX4 to inhibit ferroptosis. Targeting GPX4 to counteract the *PAX8‐AS1*/GPX4 axis may enhance the efficacy of standard chemotherapy regimens for ICC.

## 4 Experimental Section

### Cell Cultures

The ICC cell lines HuCC‐T1 and HCCC‐9810 were purchased from Cellcook Biotech (Guangzhou, China). HuCC‐T1 cells, HCCC‐9810 cells, and RBE cells were cultured in RPMI‐1640 medium (Gibco, CA, USA, 11875119). The culture medium for all cell lines contained 10% FBS (Procell, Wuhan, China, 164210), 100 U/mL penicillin, and 100 µg mL^−1^ streptomycin (Procell, Wuhan, China, PB180120), and cells were cultured in a humidified incubator with 5% CO_2_ at 37 °C.

### Establishment of Drug‐Resistant ICC Cell Lines

HuCC‐T1 and HCCC‐9810 cells were treated with one‐tenth the IC_50_ concentration of gemcitabine (MedChemexpress, NJ, USA, HY‐17026) or cisplatin (MedChemexpress, NJ, USA, HY‐17394) for three days. Afterward, the medium containing the chemotherapy drugs was removed, and the cells were cultured for seven days in complete medium. The drug treatment process was repeated with gradually increasing doses of the chemotherapy drugs until reaching ≈5 times the IC_50_ concentration. The cells were treated repeatedly with the IC_50_ concentration of gemcitabine or cisplatin for at least 180 days to maintain resistance.

### Organoid Culture

ICC tissue was obtained post‐surgery and minced in the lab. The tissue was digested at 37 °C for 30 min using the Tissue Digestion Solution (BioGenous, Hangzhou, China, K601003). Then, FBS was added to the digestion mixture to achieve a final concentration of 2%, and the mixture was filtered using a cell strainer. The filtered cells were collected and resuspended in the Organoid Culture ECM (BioGenous, Hangzhou, China, M315066). The organoid‐containing ECM was placed in droplets at the center of the bottom of a cell culture plate, allowed to solidify, and then supplemented with the complete cholangiocarcinoma organoid culture medium (BioGenous, Hangzhou, China, K2104‐LB).

### Patients and Specimens

ICC tissue from Fudan University Shanghai Cancer Center (FUSCC) was used for the generation of patient‐derived organoids (PDOs), FISH, and IF. The procedures involving human specimens in this study have been authorized by the Medical Ethics Committee of FUSCC.

### Whole Exome Sequencing

Genomic DNA was extracted from the samples, and its integrity and purity were evaluated by 1% agarose gel electrophoresis. DNA concentration and purity were measured using a Qubit 4.0 fluorometer (Thermo Fisher Scientific, Waltham, USA) and a NanoDrop One spectrophotometer (Thermo Fisher Scientific, Waltham, USA). The DNA was then sheared into random fragments using an ultrasonicator, followed by end repair, A‐tailing, and ligation to adapters containing index sequences. PCR amplification was performed using universal primers complementary to the adapter sequences. The pre‐library was hybridized with Agilent SureSelect probes in hybridization buffer, and target fragments were captured with magnetic beads, purified, and subjected to a subsequent PCR amplification to obtain the final sequencing library. The libraries were sequenced on the Illumina NovaSeq 6000 platform with 150 bp paired‐end reads.

### RNA Sequencing

Total RNAs were extracted from the samples, and its integrity and purity were evaluated by 1% agarose gel electrophoresis. Simultaneously measure RNA levels using Nanodrop One (Thermo Fisher Scientific, MA, USA). Use the Agilent 4200 system (Agilent Technologies, Waldbron, Germany) to accurately detect RNA integrity. The RNA was captured using 3′ end library preparation, followed by the addition of sequencing adapters and PCR amplification to prepare the sequencing library. The libraries were sequenced on the Illumina NovaSeq 6000 platform with 150 bp paired‐end reads.

### RNA Extraction and Quantitative Real‐Time PCR (qRT‐PCR) Analysis

Total RNA was extracted from ICC tissues or cell lines using TRIzol reagent (Invitrogen, CA, USA, 15596‐026). Reverse transcription was performed using the PrimeScript RT reagent kit (Takara, Dalian, China, RR420A). qRT‐PCR was conducted using the CFX Opus 384 PCR system (Bio‐Rad, CA, USA). The temperature cycling protocol was set according to the manufacturer's instructions. The relative RNA expression levels were normalized to *ACTB*, using the 2^‐ΔΔCT^ method. The sequences of the primers used in this study are listed in Table S3 (Supporting Information).

### Actinomycin D Assay

Cells were treated with 2 µg mL^−1^ actinomycin D (Sigma‐Aldrich, MO, USA, A1410) and were harvested at different treatment timepoints. The RNA expression levels were quantified by qRT‐PCR.

### Immunohistochemistry (IHC)

Tumor tissues or organoids were fixed in 4% paraformaldehyde and embedded in paraffin. Tissue sections were deparaffinized and rehydrated, which was followed by antigen retrieval through heat mediation in citrate buffer. Samples were blocked with 5% BSA for 1 h. Primary antibodies were incubated overnight at 4 °C, which was followed by incubation with secondary antibodies at room temperature for 1 h. Diaminobenzidine (DAB) solution was used for chromogenic reaction.

### Immunofluorescence (IF)

Samples were fixed in 4% paraformaldehyde at room temperature for 15 min, permeabilized with 0.2% Triton X‐100 for 10 min, and then blocked with 5% BSA at room temperature for 1 h. The slices were incubated overnight with primary antibodies at 4 °C and then incubated with fluorescent secondary antibodies at room temperature for 1 h. Samples were mounted after staining with diamidino‐2‐phenylindole (DAPI).

### Fluorescent In Situ Hybridization (FISH)

Specific fluorescently labeled *PAX8‐AS1* probes were designed and synthesized by RiboBio (Guangzhou, China). After fixation, permeabilization, and prehybridization, the samples were hybridized overnight with the probes in a hybridization buffer at 37 °C. The hybridization buffer was then gradually washed off with 4 × SSC (including 0.1% Tween‐20), 2 × SSC, and 1 × SSC at 42 °C. Nuclei were counterstained with DAPI. The RNA probe sequences are provided in Table S4 (Supporting Information).

### Western Blot Analysis

Proteins were isolated from ICC cells and tumor tissues using radioimmunoprecipitation assay (RIPA) buffer (Epizyme, Shanghai, China, PC101) supplemented with protease and phosphatase inhibitors. The protein concentration was determined with a bicinchoninic acid reagent (Epizyme, Shanghai, China, ZJ101). Proteins were separated using sodium dodecyl sulfate‐polyacrylamide gel electrophoresis (SDS‐PAGE) and then transferred onto polyvinylidene difluoride membranes (Merck Millipore, MA, USA, IPVH00010). After blocking the membranes in 5% skim milk for 1 h, they were incubated with primary antibodies overnight at 4 °C. The following day, the membranes were incubated with secondary antibodies at room temperature for 1 h. Target proteins were detected using the Ultrasensitive ECL Detection Kit (Proteintech, Wuhan, China, PK10003) with Tanon 5200 Imaging System (Tanon, Shanghai, China). β‐actin was used as the loading control in this study. The primary antibodies used in this study are listed in Table S5 (Supporting Information).

### Cell Viability Assay

Cells or organoids were seeded into a 96‐well plate. The following day, different concentrations of the drugs were added to each well. After 72 h of treatment, Cell Counting Kit‐8 (CCK‐8) solution (Dojindo, Kumamoto, Japan, CK04) was added to each well, and incubated for 2 h. Absorbance at 450 nm was measured using a microplate reader (Thermo Fisher Scientific, MA, US). The IC_50_ (half‐maximal inhibitory concentration) was calculated using a nonlinear regression model in GraphPad 9.0.

### Colony Formation Assay

Cells (500 cells/well) were seeded into a 12‐well plate. The following day, the cells were treated with the drug at half the IC_50_ concentration or with Dimethyl sulfoxide (DMSO) for 72 h. After removing the drug, the cells were cultured for an additional 2 weeks. Colonies were fixed with 4% paraformaldehyde and stained with 0.1% crystal violet. The number of colonies was quantified using ImageJ software.

### PI Staining

Cells (1 × 10^5^ cells/well) were seeded into a 6‐well plate. The following day, the cells were treated with the drug at half the IC_50_ concentration or with DMSO for 72 h. After treatment, the cells were collected and stained with 20 µg mL^−1^ Propidium Iodide (PI) (Sigma‐Aldrich, MO, USA, P4170) for 15 min. Following PBS washing, the cells were analyzed by flow cytometry using a CytoFlex S (Beckman Coulter, CA, USA).

### 3D Microtumor Spheroids Formation and Growth Assay

Tumor cell monolayers were washed twice with PBS then cell dissociation enzymes were added to obtain single cell suspensions without cell clusters. Cells were counted using a hemocytometer and the cell suspension was diluted according to the optimal cell density for each cell line to obtain 0.5‐2 × 10^4^ cells/ml. 200 µl/well was then dispensed into ultra‐low attachment (ULA) 96‐well round bottom plates (Corning, NY, USA, 7007) using a multichannel pipette. The plates were transferred to an incubator (37 °C, 5% CO_2_, 95% humidity). The following day, the microtumor spheroids were treated with the drug at half the IC_50_ concentration or with DMSO for 72 h. After removing the drug, the cells were cultured for an additional 5 days. Images of microtumor spheroids were acquired after culturing for different durations and the diameter was calculated.

### Live/Dead Cells Double Staining Assay

Organoids or microtumor spheroids were treated with the indicated drug or DMSO for 72 h. The samples were then washed with PBS and stained with 2 µM Calcein‐AM and 5 µM EthD‐1 (Solarbio, Beijing, China, CA1631) for 1 h at 37 °C. Following another PBS wash, images were captured using a fluorescence microscope (Leica, Wetzlar, Germany). The quantification of live and dead cells was performed by measuring the fluorescence intensity of each dye using ImageJ software.

### Reactive Oxygen Species (ROS) Assay

Cells (1 × 10^5^ cells/well) were seeded into a 6‐well plate. The following day, the cells were treated with the drug at half the IC_50_ concentration for 72 h. After treatment, the cells were collected and stained with 10 µM 2′,7′‐dichlorodihydrofluorescein diacetate (DCFH‐DA) (MedChemexpress, NJ, USA, HY‐D0940) and incubated at 37 °C for 30 min. Following PBS washing, the cells were analyzed by flow cytometry using a CytoFlex S (Beckman Coulter, CA, USA).

### Lipid Peroxidation Assay

MDA levels were assessed using the Malondialdehyde (MDA) Content Assay Kit (Solarbio, Beijing, China, BC0025). Briefly, MDA in the sample reacts with thiobarbituric acid (TBA) to form an MDA‐TBA adduct, which can be quantified colorimetrically at 532 nm. Lipid peroxidation was measured with BODIPY 581/591 C11 (MedChemexpress, NJ, USA, HY‐D1301). Cells (1 × 10^5^ cells/well) were seeded into a 6‐well plate. On the following day, cells were treated with the drug at half the IC50 concentration for 72 h. After treatment, cells were collected, stained with 5 µM BODIPY 581/591 C11, and incubated at 37 °C for 30 min. After washing with PBS, cells were analyzed by flow cytometry using a CytoFlex S (Beckman Coulter, CA, USA).

### Glutathione (GSH) Assay

GSH levels were measured using the GSH Content Assay Kit (Solarbio, Beijing, China, BC1175). Briefly, 5,5′‐dithiobis‐2‐nitrobenzoic acid reacts with intracellular GSH to form a yellow product. Absorbance was measured at 412 nm, and GSH content in cellular extracts was quantified by comparison with a calibration curve generated using GSH standards. Data were normalized to cellular protein content.

### Transmission Electron Microscopy (TEM)

Cells were treated with the drug at half the IC50 concentration for 72 h. After treatment, they were fixed for 5 min with 2.5% glutaraldehyde solution, scraped, and then collected by centrifugation to form a pellet. The pellet was fixed again with 2.5% glutaraldehyde for an additional 30 min at room temperature, and subsequently with osmium tetroxide. Following dehydration and embedding, thin sections were cut, stained with uranyl acetate and lead citrate, and examined using a HITACHI HT 7800 TEM system.

### Co‑Immunoprecipitation (Co‐IP)

The Co‐IP assays were analyzed using the Pierce Classic Magnetic IP/Co‐IP Kit (Thermo Fisher Scientific, MA, USA, 88 804). After lysing the cells with IP lysis buffer, centrifuged to collect the supernatant. Incubated the supernatant with the appropriate antibody at 4 °C overnight to form the immune complex. Incubated the immune complex with magnetic beads at room temperature for 1 h, and collected the immunoprecipitate. The immunoprecipitate was used for Western blot analysis to detect protein interactions

### RNA Pull Down Analysis

The RNA pull‐down assay was performed according to the instructions provided in the Pierce Magnetic RNA‐Protein Pull‐Down Kit (Thermo Scientific, MA, USA, 20 164). *PAX8‐AS1* RNA was transcribed in vitro using a DNA template containing the T7 promoter, following the instructions for the Ribo RNAmax‐T7 Transcription Kit (RiboBio, Guangzhou, China, C11001). *PAX8‐AS1* was then end‐labeled with desthiobiotin using the Pierce RNA 3′ End Desthiobiotinylation Kit (Thermo Scientific, MA, USA, 20 163). Biotinylated *PAX8‐AS1* was captured with streptavidin magnetic beads and subsequently incubated with cell extract. The protein was eluted from the RNA‐protein complex, and after SDS‐PAGE, the gel was stained using the Fast Silver Stain Kit (Beyotime, Beijing, China, P0017S). Mass spectrometry was performed to identify RNA‐binding proteins in the target region.

### RNA Immunoprecipitation (RIP) Assay

The RIP assay was performed using a Magna RIP RNA‐binding protein immunoprecipitation kit (Merck Millipore, MA, USA, 17–700) according to the manufacturer's protocol. In brief, cells were lysed in RIP lysis buffer, and the lysate was incubated overnight at 4 °C with antibody‐conjugated beads. Next, RNA was purified and subjected to qRT‐PCR to assess gene expression.

### Luciferase Reporter Assay

Fragments of the antioxidant response element (ARE) or *GPX4* promoter were cloned into the PGL3 luciferase plasmid and co‐transfected into cells along with the specified plasmids. At 48 h after transfection, the luciferase activities were detected using the Dual‐Luciferase Reporter Assay System (Promega, WI, USA, E1910) according to the manufacturer's protocol. The luciferase activities were normalized to the corresponding Renilla luciferase activities.

### Fluorescence Recovery After Photobleaching (FRAP) Assay

FRAP assays were performed on an Olympus FV3000 confocal microscope. Puncta were photobleached for 1 s using a 488 nm laser at 70% laser power. After bleaching, time‐series images were acquired every 1 s. The recovery from photobleaching was recorded for the indicated time. The recovery curves were analyzed using ImageJ software.

### Protein Expression and Purification

The 6 × His‐tagged p62 gene was cloned into the pET‐28a vector (Novagen, Germany) and transformed into *Escherichia coli* BL21(DE3) cells for recombinant protein expression induced by 0.2 mM isopropyl β‐D‐1‐thiogalactopyranoside (IPTG). Cells were harvested, resuspended in binding buffer (20 mM Tris, 0.5 M NaCl, 5 mM imidazole, pH 7.9) supplemented with protease inhibitor cocktail and 0.1 mg/ml phenylmethylsulfonyl fluoride (PMSF), then lysed using BugBuster 10 × protein extraction reagent (1:10 v/v) and 50 U/ml Benzonase nuclease. After centrifugation at 16000 × g for 20 min, the supernatant was incubated with Ni‐NTA affinity resin (Novagen, Germany), followed by sequential washes with binding buffer and wash buffer (20 mM Tris‐HCl, 0.5 M NaCl, 60 mM imidazole, pH 7.9). Bound proteins were eluted with elution buffer (0.5 M NaCl, 20 mM Tris, 1 M imidazole, pH 7.9) and further purified by size‐exclusion chromatography using a Superose 6 Increase 10/300 GL column (Cytiva, US), then concentrated to ≥10 µM using Amicon Ultra‐0.5 mL filters (Merck Millipore, MA, US).

### In Vitro Phase Separation Assays

The LLPS assay of p62 was conducted in 384‐well plates. Purified p62 protein was incubated with specified concentrations of *PAX8‐AS1* in a reaction buffer (40 mM Tris‐HCl [pH 7.4], 150 mM NaCl, 1 mM DTT) at room temperature for 15 min, followed by image acquisition.

### *N* ^6^‐Methyladenosine (m^6^A) RNA Methylation Quantification Assay

Total m^6^A levels were assessed using the EpiQuik m^6^A RNA Methylation Quantification Kit (Epigentek, NY, USA, P‐9005). Briefly, total cellular RNA was extracted, and 200 ng of the RNA was added to each well for binding. Subsequently, capture antibodies and detection antibodies were added to the wells to detect m^6^A. The amount of m^6^A was proportional to the optical density (OD) measured at 450 nm. Total m^6^A levels were determined by applying the formula provided with the kit.

### Animal Experiments

Six‐week‐old nude mice were purchased from Sinogene Biotech (Shanghai, China). For the subcutaneous xenograft model, HuCC‐T1 cells (5 × 10^6^ cells per mouse) were subcutaneously injected into nude mice. The tumor size was recorded every two days, and the volume was calculated as follows: volume (mm^3^) = (L × W^2^)/2, where L is the long axis and W is the short axis. Once the tumors reached a volume of ≈100 mm^3^, the mice were randomly divided into different groups and treated with saline, gemcitabine (intraperitoneally, 10 mg/kg, three times a week), or cisplatin (intraperitoneally, 2 mg/kg, three times a week). Five weeks later, the mice were euthanized, and their tumors were isolated and weighed.

For the orthotopic tumor model, HuCC‐T1 cells stably expressing luciferase (5 × 10^6^ cells per mouse) were implanted into the livers of nude mice under anesthesia. After four weeks, the mice were randomly divided into different groups and received the same treatments as in the subcutaneous model. After five weeks of treatment, tumor growth was visualized using the IVIS system following a D‐luciferin injection.

In the JKE‐1674 combined chemotherapy treatment, after the establishment of subcutaneous and orthotopic tumor models, mice were randomized into different treatment groups. They were treated with either saline; gemcitabine (intraperitoneally, 10 mg/kg, three times a week, MedChemExpress, HY‐17026); cisplatin (intraperitoneally, 2 mg/kg, three times a week, MedChemExpress, HY‐17394); JKE‐1674 (orally, 25 mg/kg, three times a week, MedChemExpress, HY‐138153); or a combination of JKE‐1674 (orally, 25 mg/kg, three times a week) with gemcitabine (intraperitoneally, 10 mg/kg, three times a week) or cisplatin (intraperitoneally, 2 mg/kg, three times a week). For the PDOX model, 5 × 10^4^ PDOs suspended in 50 µl of Matrigel were subcutaneously injected into nude mice. Once the tumors reached a volume of ≈100 mm^3^, the mice were randomly assigned to different treatment groups. They received either saline; gemcitabine (intraperitoneally, 10 mg/kg, three times a week) plus cisplatin (intraperitoneally, 2 mg/kg, three times a week); JKE‐1674 (orally, 25 mg/kg, three times a week); or a combination of JKE‐1674 (orally, 25 mg/kg, three times a week) with gemcitabine (intraperitoneally, 10 mg/kg, three times a week) and cisplatin (intraperitoneally, 2 mg/kg, three times a week). After eight weeks of treatment, the antitumor efficacy of each regimen was assessed. All animal studies were approved by the Institutional Animal Care and Use Committee of Fudan University.

### Statistical Analysis

GraphPad Prism 9.0 and R 4.4.1 were applied for statistical analysis. Data between two groups were assessed using Student's *t‐*test. Data in multiple groups were evaluated using analysis of variance (ANOVA). The Mann–Whitney U test was used for the comparison of non‐normal distributed data. Overall survival (OS) was assessed with the Kaplan−Meier method and compared by the log‐rank test. Representative data are shown as the mean ± standard error of the means (SEM). A *p* value less than 0.05 was considered to be statistically significant. *p < 0.05, **p < 0.01, ***p < 0.001, and *p* ≥ 0.05 means not significant (ns).

## Conflict of Interest

The authors declare no conflict of interest.

## Author Contributions

Z.‐W.C., J.‐J.S. contributed equally to this work. L.W., L.‐R.W. and Z.‐H.L. designed the research and directed the work. Z.‐W.C. and J.‐J.S. performed the experiments and wrote the paper. Z.‐W.C., J.‐J.S. and M.C. participated in data analysis. Z.W., H.‐X.Z. and X.J. assisted with experiments. Y.‐M.Z., Y.‐X.W., Y.‐B.W., Z.X. and Z.‐W.D. provided technical help. All authors have read and approved the final manuscript.

## Supporting information



Supporting Information

Supporting Information

Supporting Information

## Data Availability

The data that support the findings of this study are available from the corresponding author upon reasonable request.
