# Supplementary material for: Targeting GPX4 to Induce Ferroptosis Overcomes Chemoresistance Mediated by the PAX8‐AS1/GPX4 Axis in Intrahepatic Cholangiocarcinoma
Source: Adv Sci (Weinh). 2025 May 20;12(30):e01042. doi: 10.1002/advs.202501042 (PMC12376697; doi:10.1002/advs.202501042)
Supplement: Supplementary file 1 — Supporting Information [file ADVS-12-e01042-s002.docx]

**Supplementary Information**

**Targeting GPX4 to Induce Ferroptosis Overcomes Chemoresistance Mediated by the *PAX8-AS1*/GPX4 Axis in Intrahepatic Cholangiocarcinoma**

1. **Supplementary Figures:**

**Supplementary Figure 1** Establishment of organoids from ICC patients.

**Supplementary Figure 2** *PAX8-AS*1 promotes chemoresistance in ICC cells.

**Supplementary Figure 3** *PAX8-AS1* has no effect on the proliferation and death of ICC cells.

**Supplementary Figure 4** *PAX8-AS1* promotes chemotherapy resistance in organoids and *in vivo*.

**Supplementary Figure 5** *PAX8-AS1* does not affect cell growth and death in organoids and *in vivo*.

**Supplementary Figure 6** *PAX8-AS1* regulates the KEAP1/NRF2 pathway to inhibit ferroptosis.

**Supplementary Figure 7** Western blot analysis of KEAP1 in the indicated groups.

**Supplementary Figure 8** The negative results related to Figure 7.

**Supplementary Figure 9** Validation of biosafety and GEPIA database analysis.

1. **Supplementary Tables (refer to the corresponding supplementary file):**

**Supplementary Table 1** Baseline clinical characteristics of chemotherapy-treated patients in the TCGA_CHOL dataset.

**Supplementary Table 2** *PAX8-AS1*-interacting proteins identified by RNA pull-down mass spectrometry and predicted by ENCORI, RBPmap, and RNAInter.

**Supplementary Table 3** Primer sequences used in this study.

**Supplementary Table 4** Sequences of the shRNA, sgRNA, and FISH probe used in this study.

**Supplementary Table 5** Primary antibodies used in this study.

**
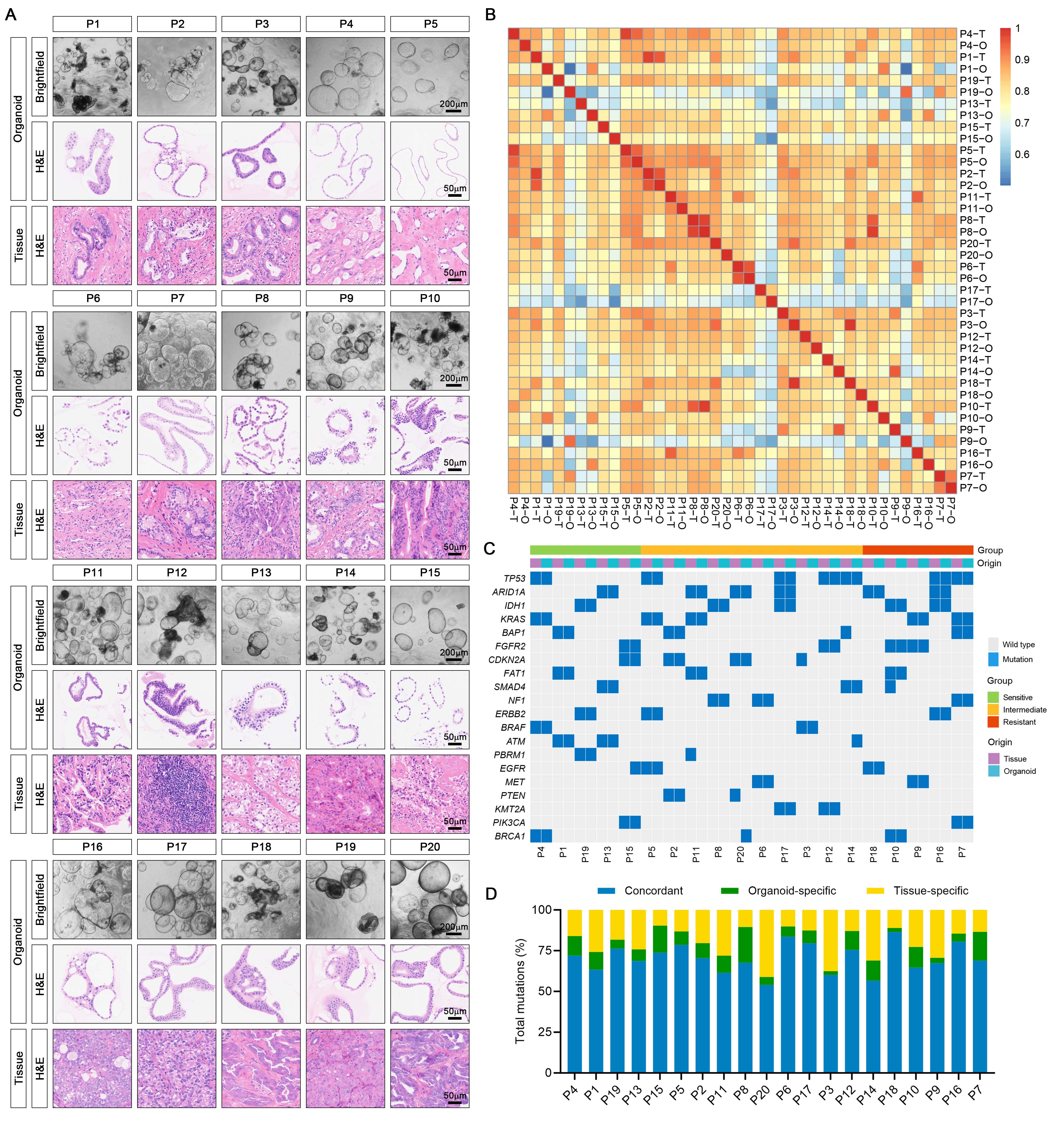
**

**Supplementary Figure 1 Establishment of organoids from ICC patients. (A)** Representative bright-field and H&E microscopic images of organoids and corresponding tissues derived from 20 ICC patients. **(B)** Correlation heatmap of expression profiles between parental tumors and matched organoids based on RNA-seq data. **(C)** Top 20 mutated genes identified by WES in primary tumors and corresponding organoids. **(D)** Concordance of the mutations detected between the primary tumors and corresponding organoids.

**
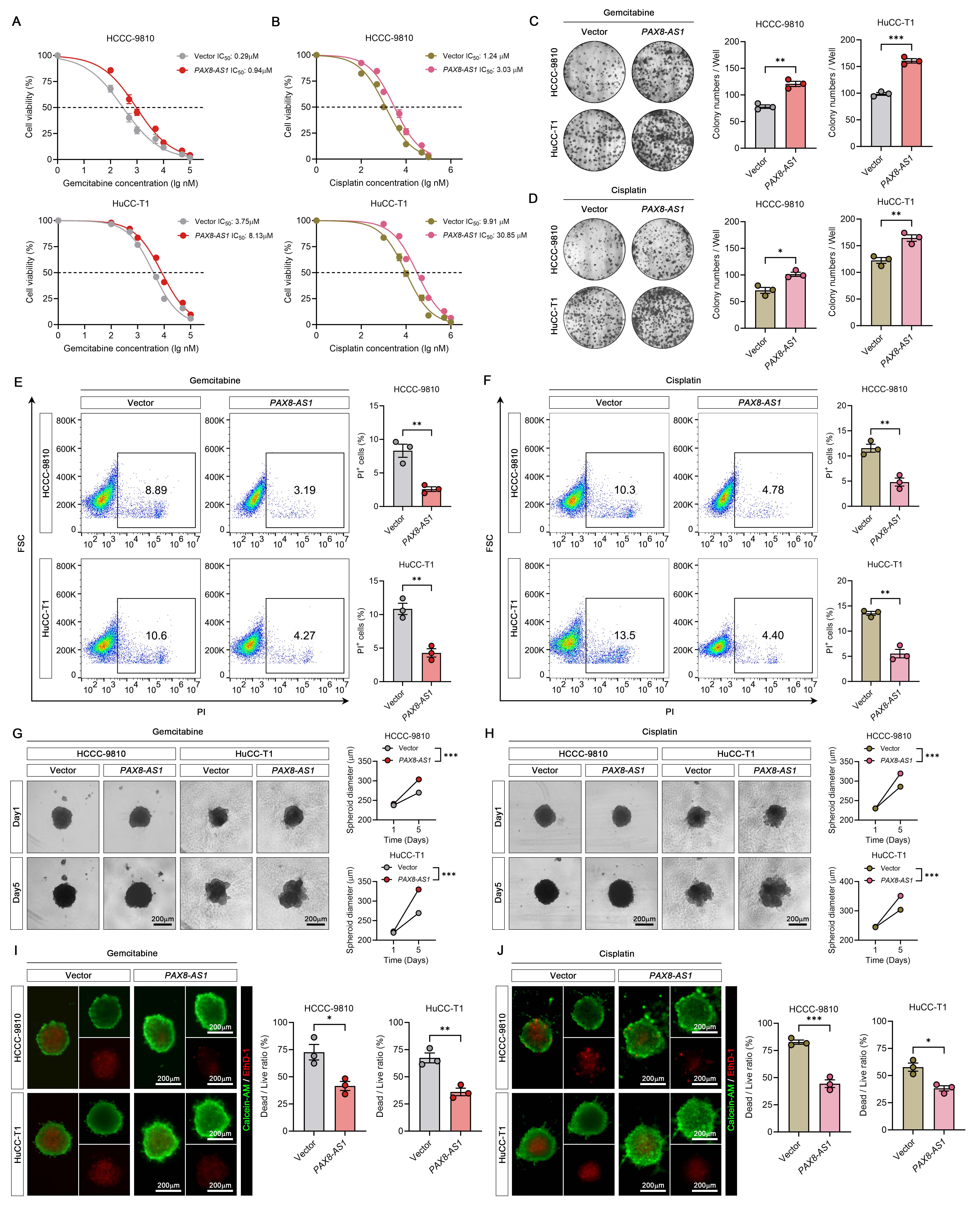
**

**Supplementary Figure 2 *PAX8-AS*1 promotes chemoresistance in ICC cells. (A, B)** Dose-response curves for gemcitabine and cisplatin in ICC cells with overexpression of *PAX8-AS1*; *n* = 3 biologically independent samples. **(C, D)** Representative images and statistical analysis of colony formation in the indicated cells treated with gemcitabine or cisplatin at half the IC_50_ concentration for 72 hours, followed by a two-week incubation; *n* = 3 biologically independent samples. **(E, F)** Flow cytometry images and statistical analysis of PI-stained cells after 72 hours of treatment with gemcitabine or cisplatin at half the IC_50_ concentration; *n* = 3 biologically independent samples. **(G, H)** Representative images of ICC microtumor spheroid and quantification of their diameters on day one and day five after treatment with gemcitabine or cisplatin at half the IC_50_ concentration for 72 hours; *n* = 3 biologically independent samples. **(I, J)** Representative images and fluorescence intensity ratios between dead (Eth-D1) and live (Calcein-AM) cells in ICC microtumor spheroids after 72 hours of treatment with gemcitabine or cisplatin at half the IC_50_ concentration; *n* = 3 biologically independent samples. Data are the mean ± SEM. **P* < 0.05, ***P* < 0.01, ****P* < 0.001. *P* values were determined by unpaired two-tailed Student’s *t*-tests (C-F, I, J) and two-way ANOVA (G, H).

**
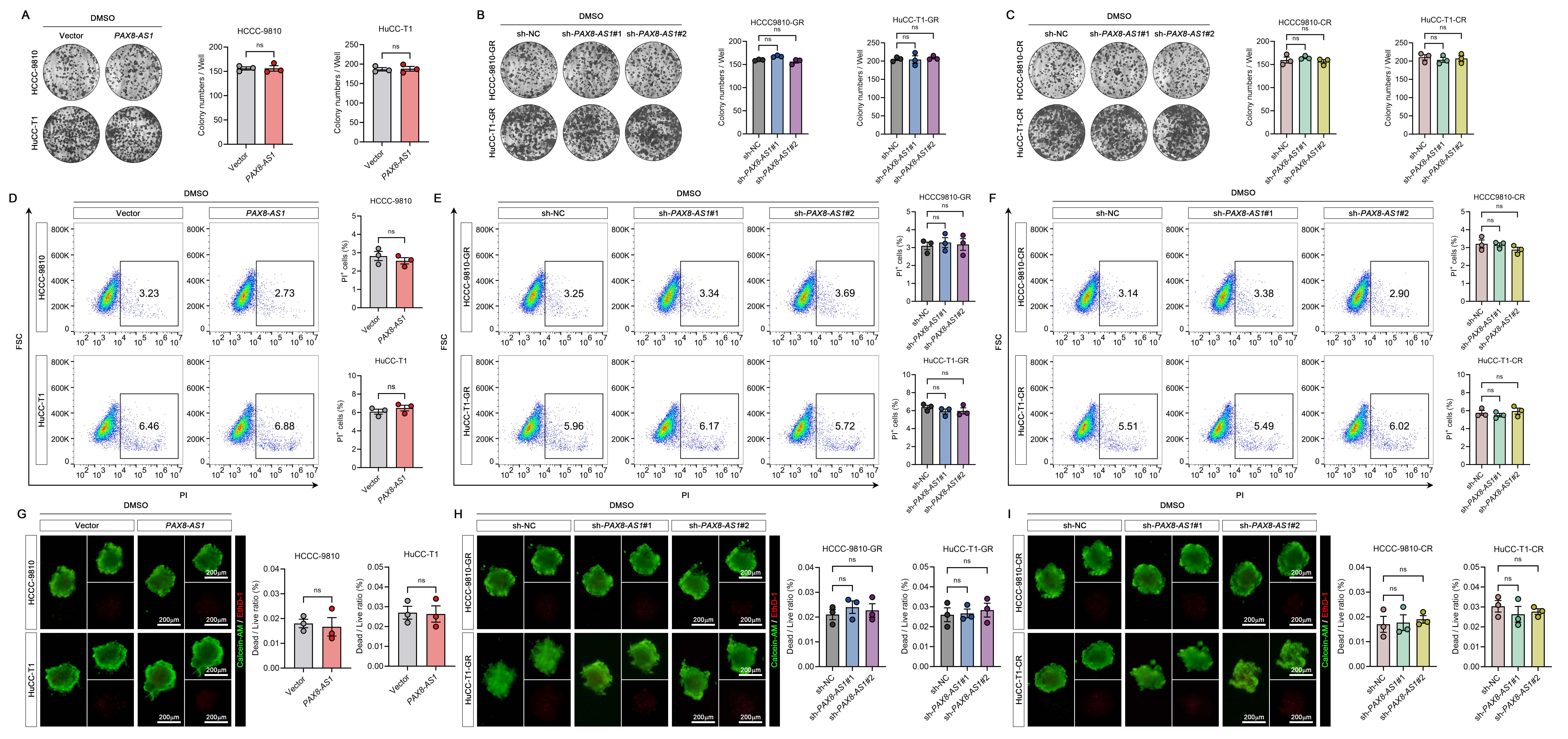
Supplementary Figure 3 *PAX8-AS1* has no effect on the proliferation and death of ICC cells. (A-C)** Representative images and statistical analysis of colony formation in the indicated cells treated with DMSO for 72 hours, followed by a two-week incubation; *n* = 3 biologically independent samples. **(D-F)** Representative flow cytometry images and statistical analysis of PI-stained cells after 72 hours of treatment with DMSO; *n* = 3 biologically independent sample. **(G-I)** Representative images and fluorescence intensity ratios between dead (Eth-D1) and live (Calcein-AM) cells in ICC microtumor spheroids after 72 hours of treatment with DMSO; *n* = 3 biologically independent sample. Data are the mean ± SEM. ns, not significant. *P* values were determined by unpaired two-tailed Student’s *t*-tests (A, D, G) and one-way ANOVA (B, C, E, F, H, I).


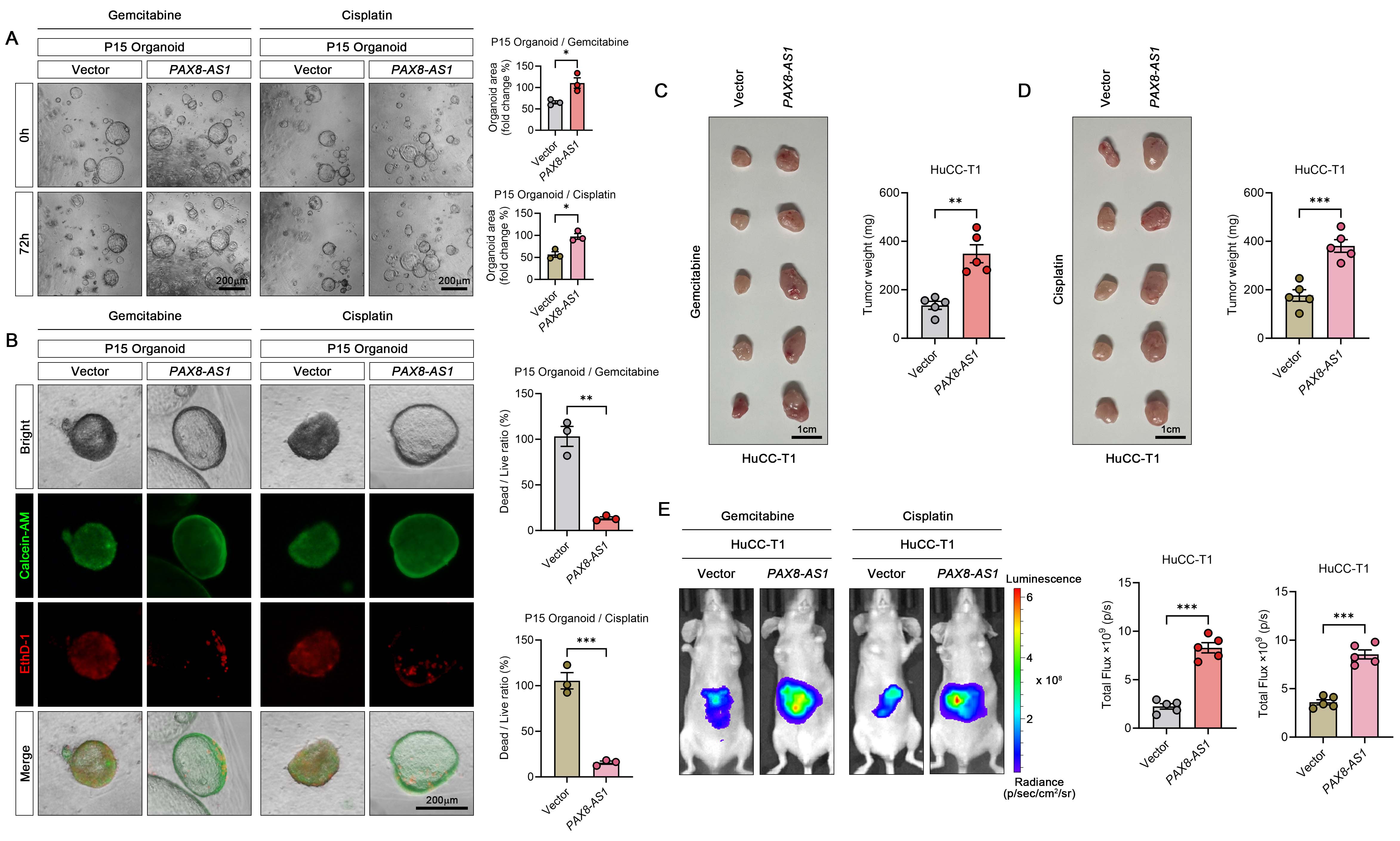


**Supplementary Figure 4 *PAX8-AS1* promotes chemotherapy resistance in organoids and *in vivo*. (A)** Representative images and statistical analysis of PDOs treated with gemcitabine (5 µM) or cisplatin (10 µM) for 72 hours; *n* = 3 biologically independent sample. **(B)** Representative images and fluorescence intensity ratios of dead (Eth-D1) versus live (Calcein-AM) cells in PDOs after 72 hours of treatment with gemcitabine (5 µM) or cisplatin (10 µM); *n* = 3 biologically independent sample. **(C, D)** Images and weights of subcutaneous xenografts from the indicated ICC cells; *n* = 5 mice per group. **(E)** Representative images and statistical analysis of *in vivo* bioluminescence in the indicated orthotopic tumor models; *n* = 5 mice per group. Data are the mean ± SEM. **P* < 0.05, ***P* < 0.01, ****P* < 0.001. *P* values were determined by unpaired two-tailed Student’s *t*-tests (A-E).

**
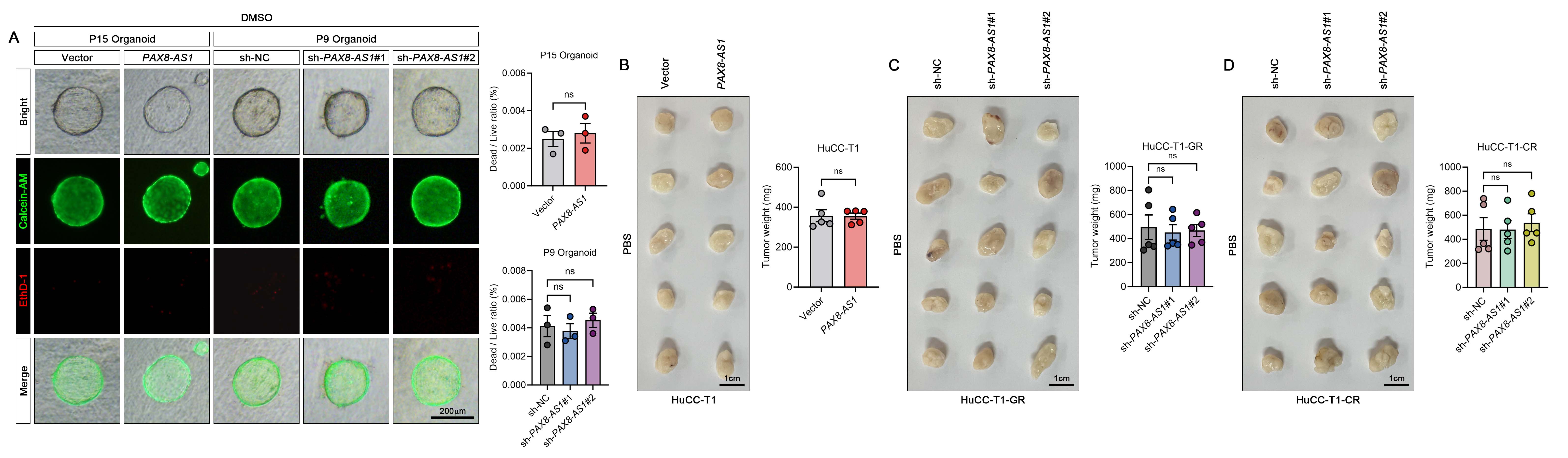
Supplementary Figure 5 *PAX8-AS1* does not affect cell growth and death in organoids and *in vivo*. (A)** Representative images and fluorescence intensity ratios of dead (Eth-D1) versus live (Calcein-AM) cells in PDOs after 72 hours of treatment with DMSO; *n* = 3 biologically independent sample. **(E-H)** Images and weights of subcutaneous xenografts from the indicated ICC cells; *n* = 5 mice per group. Data are the mean ± SEM. ns, not significant. *P* values were determined by unpaired two-tailed Student’s *t*-tests (A [upper], B) and one-way ANOVA (A [lower], C, D).

**
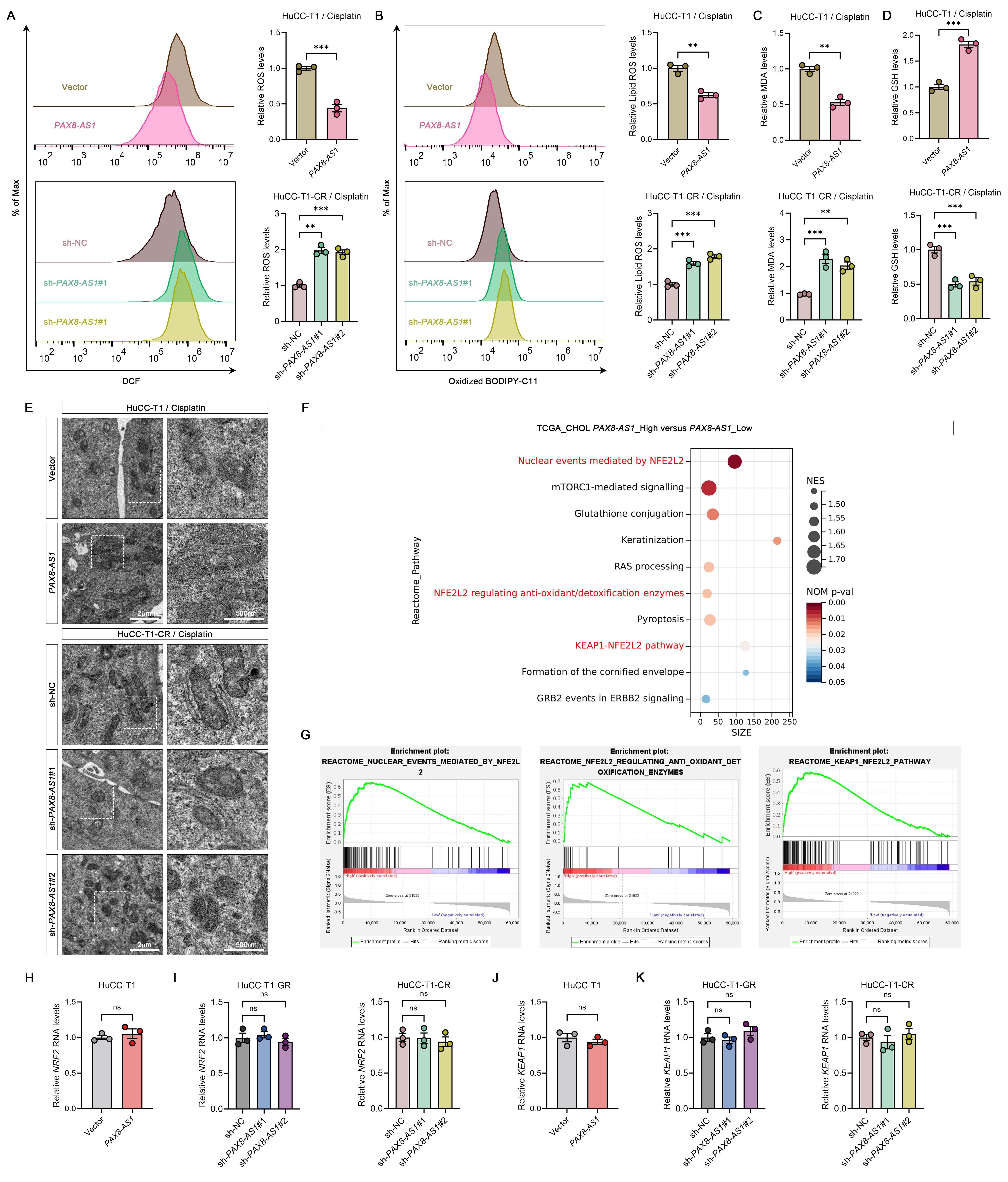
Supplementary Figure 6 *PAX8-AS1* regulates the KEAP1/NRF2 pathway to inhibit ferroptosis. (A)** Representative flow cytometry images and statistical analysis of ROS levels in the indicated cells after treatment with cisplatin (at half the IC_50_ concentration) for 72 hours; *n* = 3 biologically independent samples. **(B)** Representative flow cytometry images and statistical analysis of lipid peroxidation levels in the indicated cells after treatment with cisplatin (at half the IC_50_ concentration) for 72 hours; *n* = 3 biologically independent samples. **(C)** Levels of lipid oxidation product MDA in the indicated cells after treatment with cisplatin (at half the IC_50_ concentration) for 72 hours; *n* = 3 biologically independent samples. **(D)** GSH levels in the indicated cells after treatment with cisplatin (at half the IC_50_ concentration) for 72 hours; *n* = 3 biologically independent samples. **(E)** Representative TEM images of mitochondrial morphology and ultrastructure in the indicated cells after 72 hours of treatment with gemcitabine (at half the IC_50_ concentration); *n* = 3 biologically independent samples. **(F, G)** GSEA analysis revealed significantly enriched reactome pathways in the TCGA_CHOL *PAX8-AS1* high-expression group. **(H, I)** *NRF2* mRNA levels in cells overexpressing or knocking down *PAX8-AS1* analyzed by qPCR; *n* = 3 biologically independent samples. **(J, K)** *KEAP1* mRNA levels in cells overexpressing or knocking down *PAX8-AS1* analyzed by qPCR; *n* = 3 biologically independent samples. Data are the mean ± SEM. ***P* < 0.01, ****P* < 0.001; ns, not significant. *P* values were determined by unpaired two-tailed Student’s *t*-tests (A [upper], B [upper], C [upper], D [upper], H, J) and one-way ANOVA (A [lower], B [lower], C [lower], D [lower], I, K).


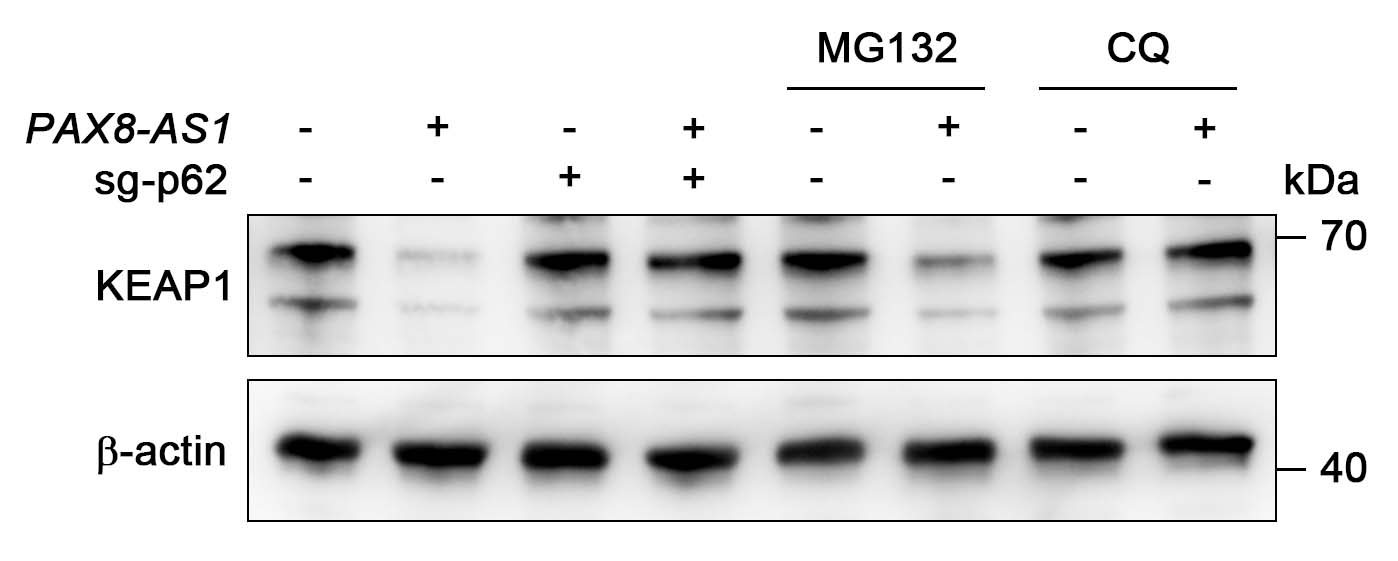


**Supplementary Figure 7 Western blot analysis of KEAP1 in the indicated groups.** Western blot analysis of KEAP1 in HuCC-T1 cells overexpressing *PAX8-AS1*, following either p62 knockout or treatment with chloroquine (CQ, 25 µM ) or MG132 (10 µM); *n* = 3 biologically independent samples.

**
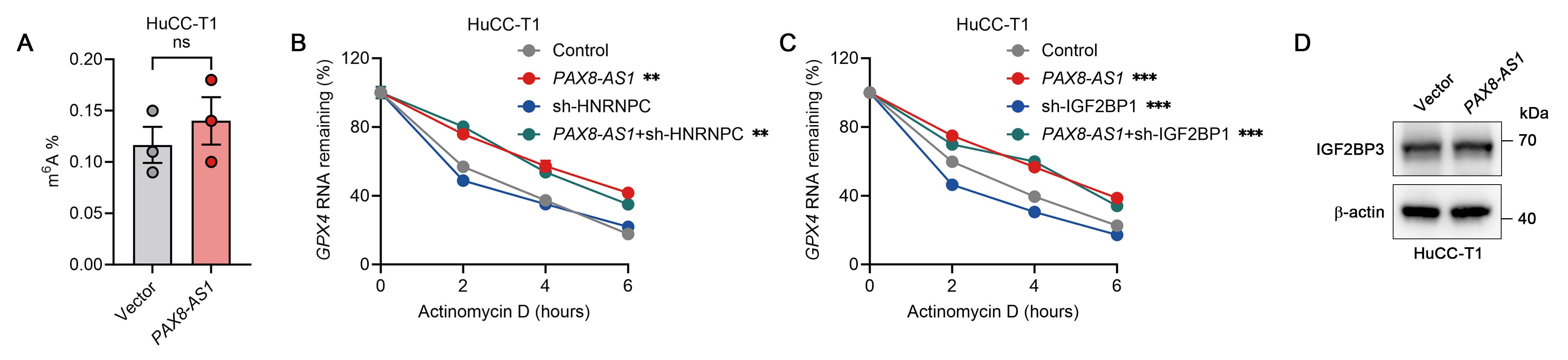
**

**Supplementary Figure 8 The negative results related to Figure 7. (A)** Overall m^6^A levels in HuCC-T1 cells after *PAX8-AS1* overexpression; *n* = 3 biologically independent samples. **(B, C)** qRT-PCR analysis of *GPX4* mRNA levels in the indicated cells after treatment with Actinomycin D (2 µg/mL) for different durations; *n* = 3 biologically independent samples. **(D)** Western blot analysis of IGF2BP3 in HuCC-T1 cells after *PAX8-AS1* overexpression; *n* = 3 biologically independent samples. Data are the mean ± SEM. ***P* < 0.01, ****P* < 0.001; ns, not significant. *P* values were determined by unpaired two-tailed Student’s *t*-tests (A) and one-way ANOVA (B, C).

**
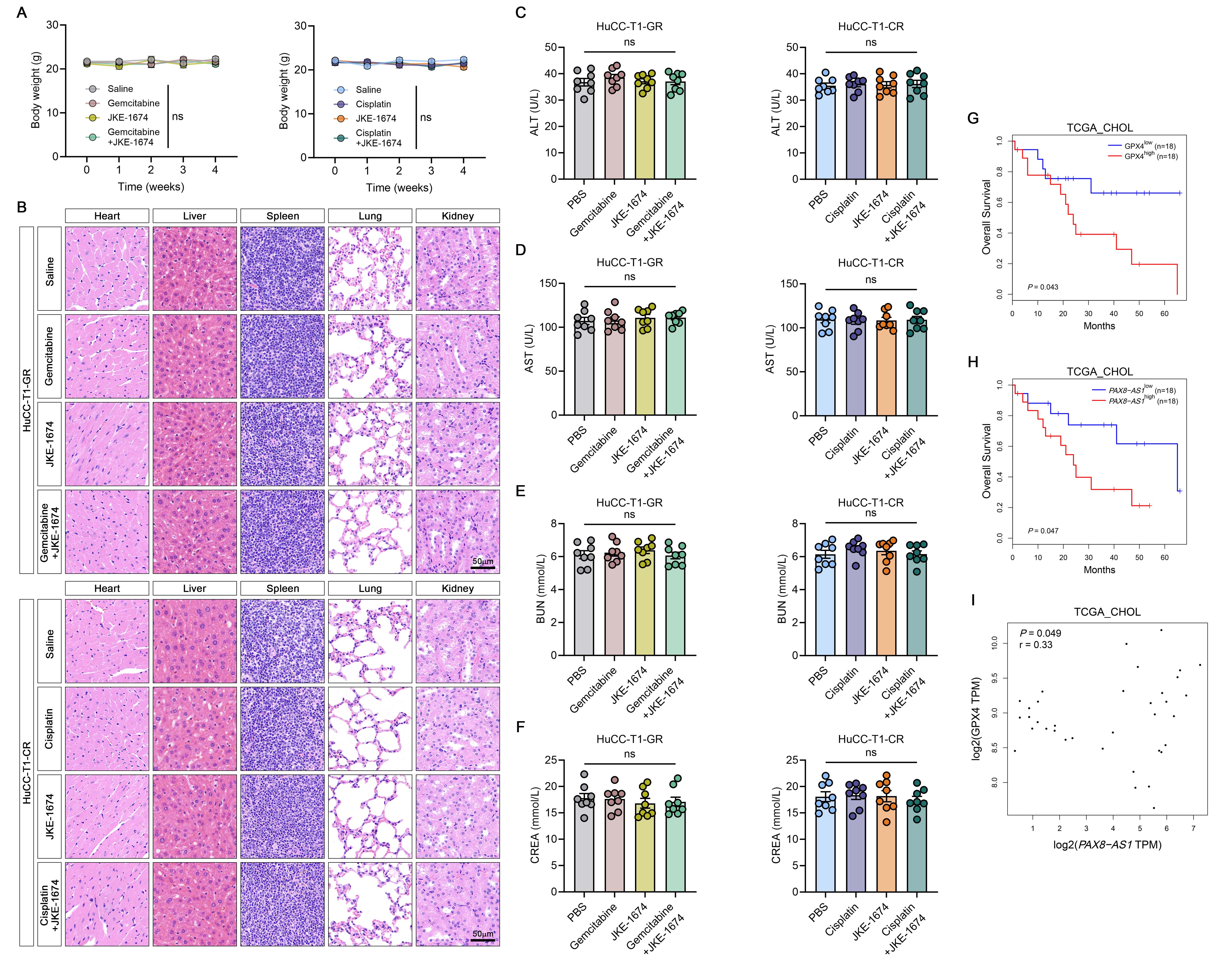
Supplementary Figure 9 Validation of biosafety and GEPIA database analysis. (A)** The body weights of subcutaneous tumor-bearing mice after the indicated treatments; *n* = 8 mice per group. **(B)** Representative H&E-stained images of the main organs from subcutaneous tumor-bearing mice after the indicated treatments; *n* = 8 mice per group. **(C-F)** The liver function (ALT and AST levels; C and D) and kidney function (BUN and CREA levels; E and F) of subcutaneous tumor-bearing mice after the indicated treatments; *n* = 8 mice per group. **(G, H)** According to the GEPIA database, survival curves for overall survival of patients in high and low expression groups of *PAX8-AS1* and GPX4 in TCGA_CHOL. **(I)** According to the GEPIA database, the correlation between *PAX8-AS1* levels and GPX4 levels in TCGA_CHOL. Data are the mean ± SEM. ns, not significant. *P* values were determined by one-way (C-F) or two-way (A) ANOVA.
