# Supplementary material for: Targeting GPX4 to Induce Ferroptosis Overcomes Chemoresistance Mediated by the PAX8‐AS1/GPX4 Axis in Intrahepatic Cholangiocarcinoma
Source: Adv Sci (Weinh). 2025 May 20;12(30):e01042. doi: 10.1002/advs.202501042 (PMC12376697; doi:10.1002/advs.202501042)

ADVANCED SCIENCE
Open Access

Supporting Information

for *Adv. Sci.*, DOI 10.1002/advs.202501042

Targeting GPX4 to Induce Ferroptosis Overcomes Chemoresistance Mediated by the *PAX8-AS1*/GPX4 Axis in Intrahepatic Cholangiocarcinoma

Zhi-Wen Chen, Ji-Jun Shan, Mo Chen, Zong Wu, Yi-Ming Zhao, Hong-Xu Zhu, Xin Jin, Yi-Xiu Wang, Yi-Bin Wu, Zhen Xiang, Zhi-Wen Ding, Zhen-Hai Lin, Long-Rong Wang* and Lu Wang**

Figure 3

L

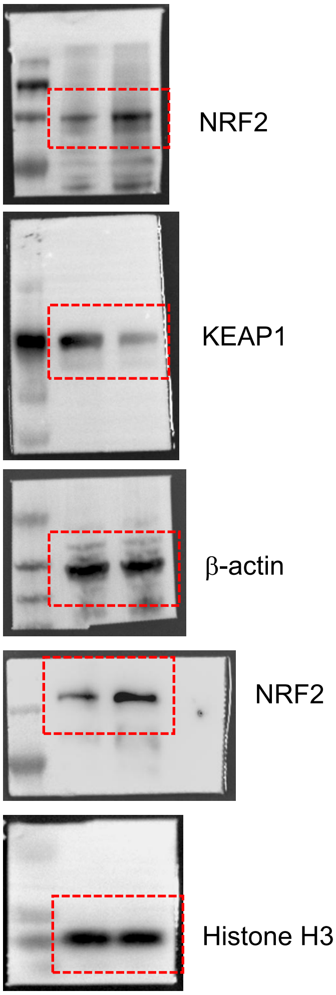
NRF2
KEAP1
β-actin
NRF2
Histone H3

M

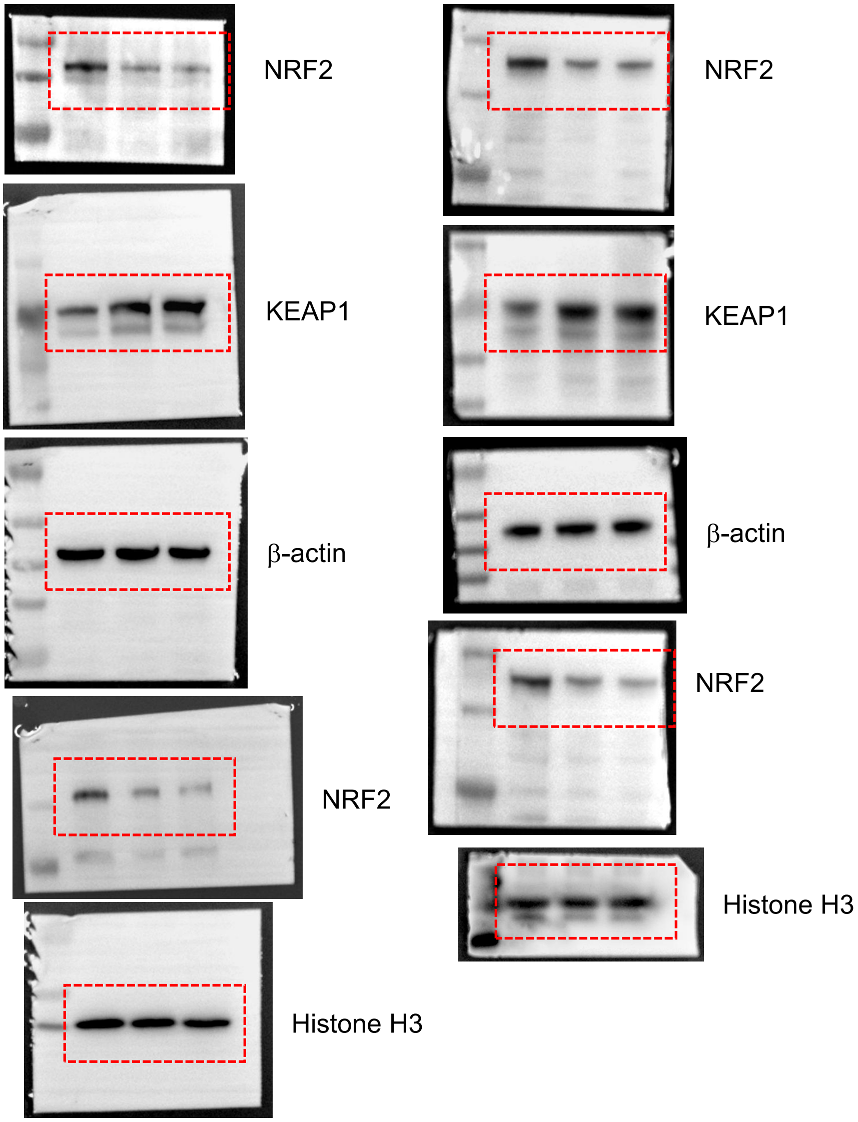
NRF2
KEAP1
β-actin
NRF2
Histone H3
NRF2
KEAP1
β-actin
NRF2
Histone H3

Figure 4

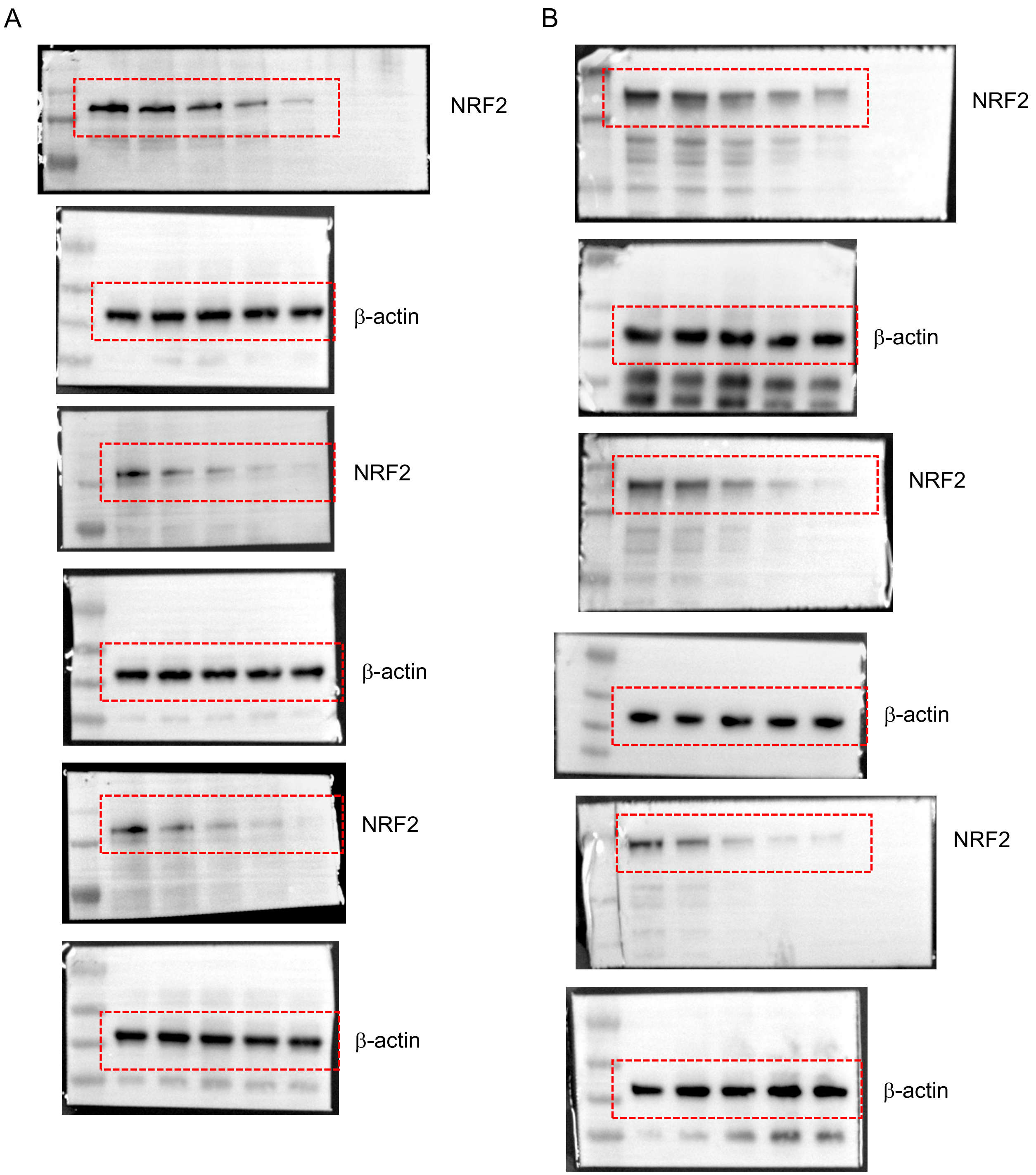
A
NRF2
β-actin
NRF2
β-actin
NRF2
β-actin
B
NRF2
β-actin
NRF2
β-actin
NRF2
β-actin

Figure 4

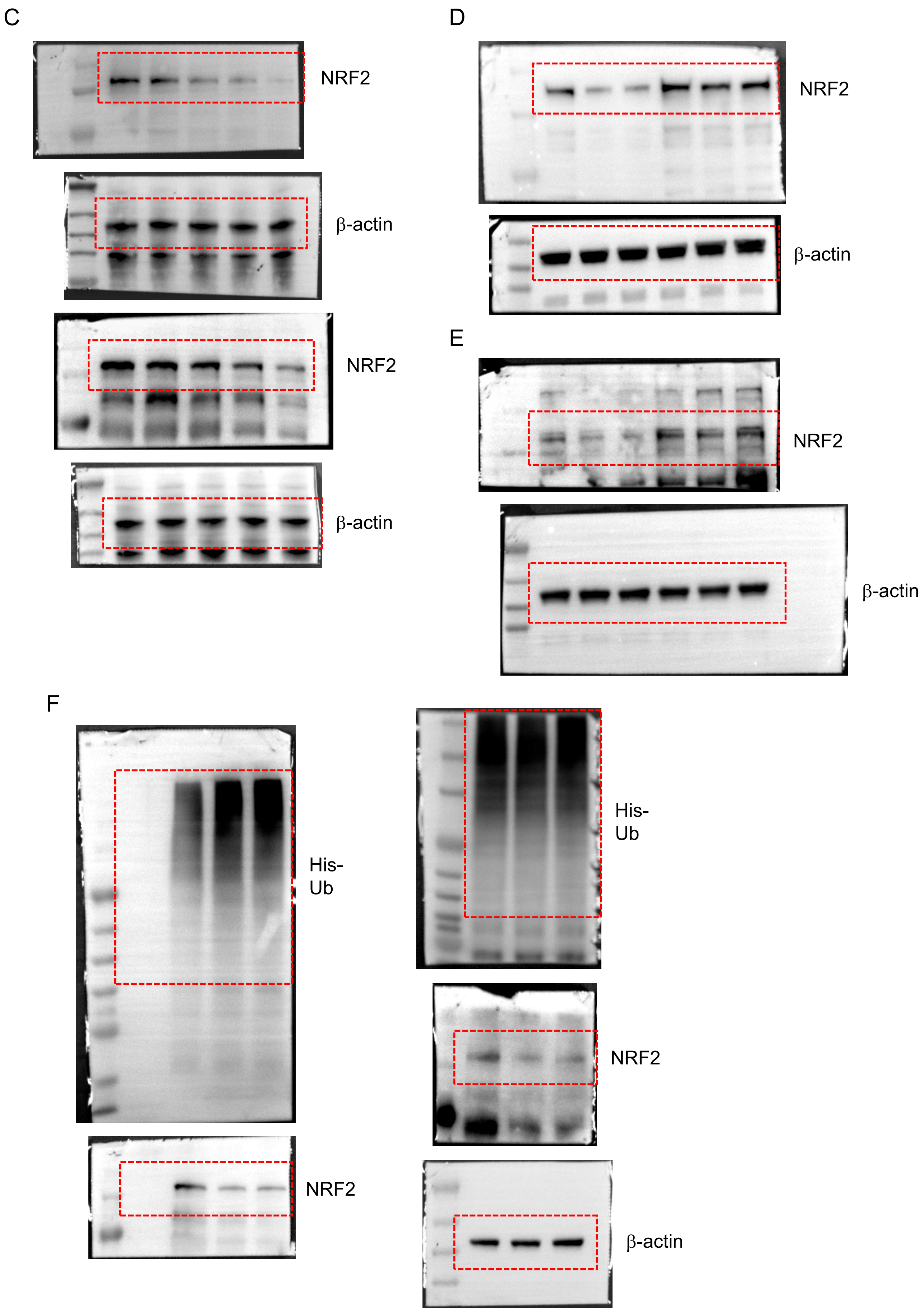
C
NRF2
β-actin
NRF2
β-actin
D
NRF2
β-actin
E
NRF2
β-actin
F
His-Ub
NRF2
His-Ub
NRF2
β-actin

Figure 4

G

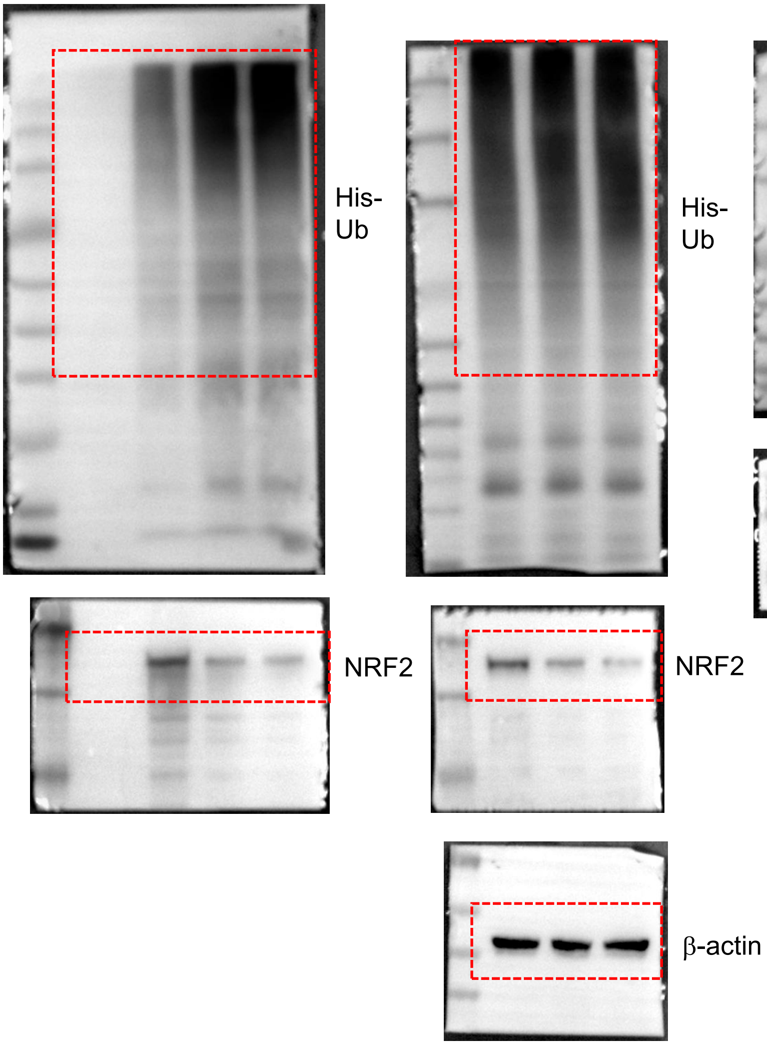

H

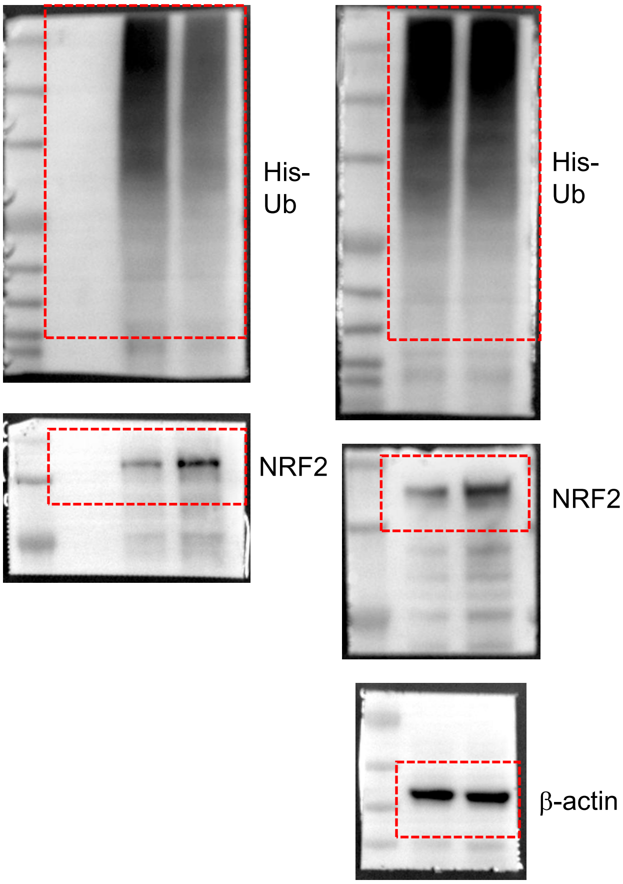

K

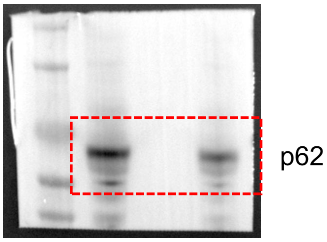

L

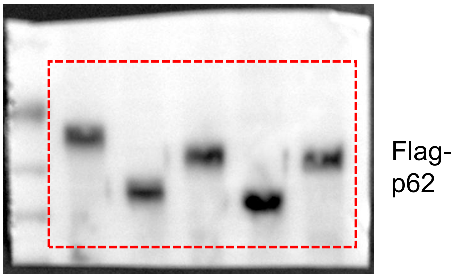

M

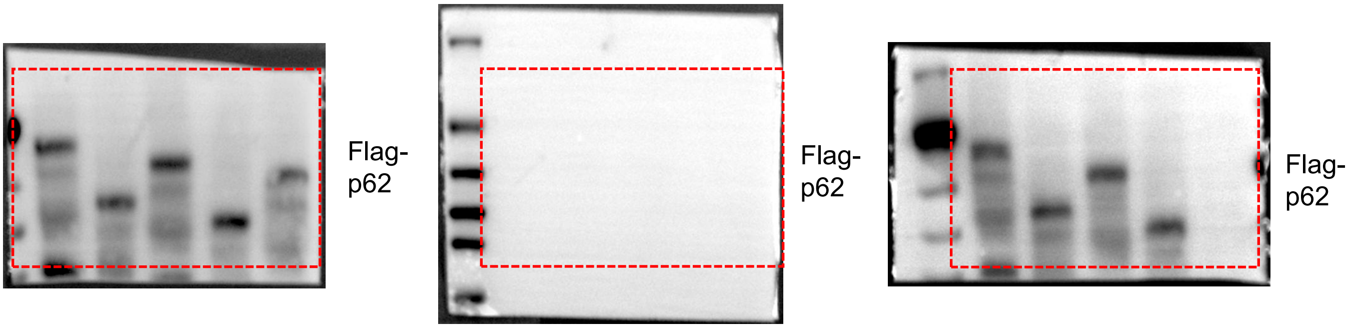

Figure 4

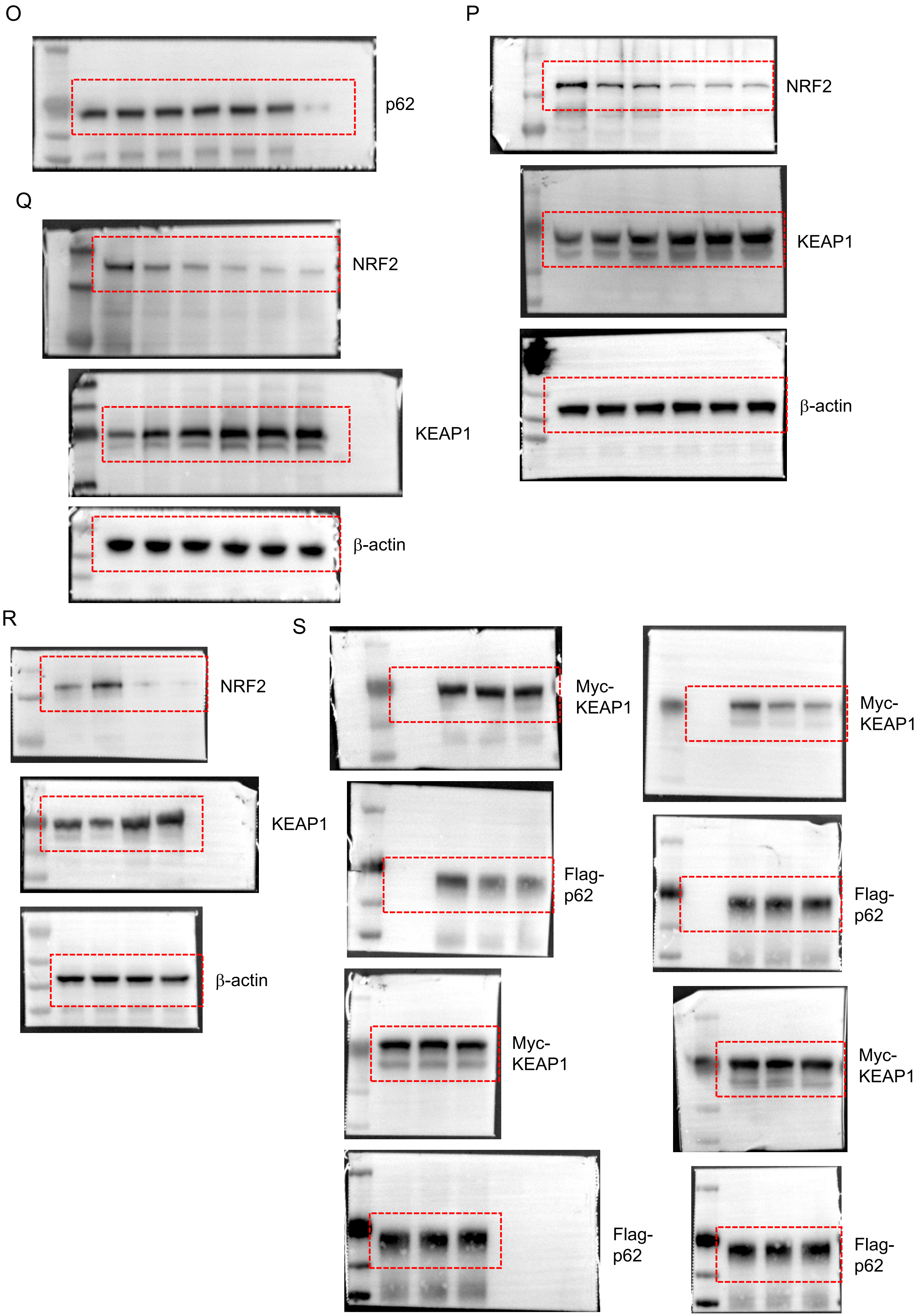

O
p62
P
NRF2
KEAP1
β-actin
Q
NRF2
KEAP1
β-actin
R
NRF2
KEAP1
β-actin
S
Myc-KEAP1
Myc-KEAP1
Flag-p62
Flag-p62
Myc-KEAP1
Myc-KEAP1
Flag-p62
Flag-p62

Figure 4

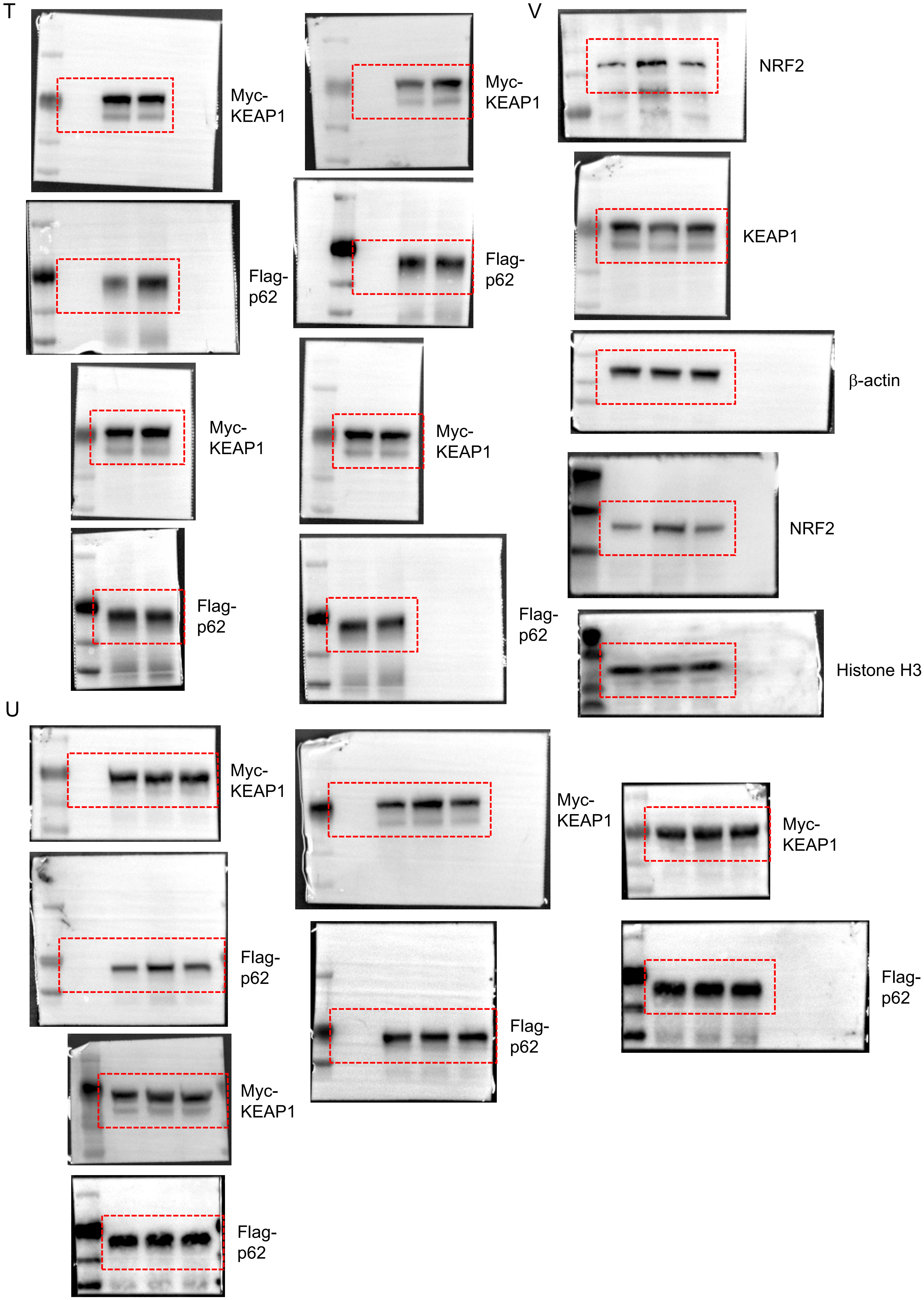
T
Myc-KEAP1
Flag-p62
Myc-KEAP1
Flag-p62
Myc-KEAP1
Flag-p62
Myc-KEAP1
Flag-p62
U
Myc-KEAP1
Flag-p62
Myc-KEAP1
Flag-p62
Myc-KEAP1
Flag-p62
Myc-KEAP1
Flag-p62
V
NRF2
KEAP1
β-actin
NRF2
Histone H3

Supplementary Figure 7

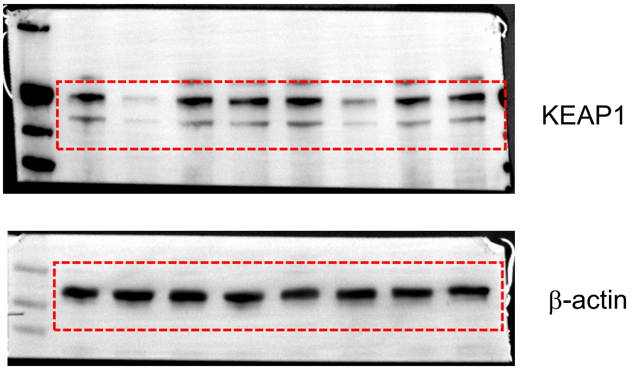

Figure 5

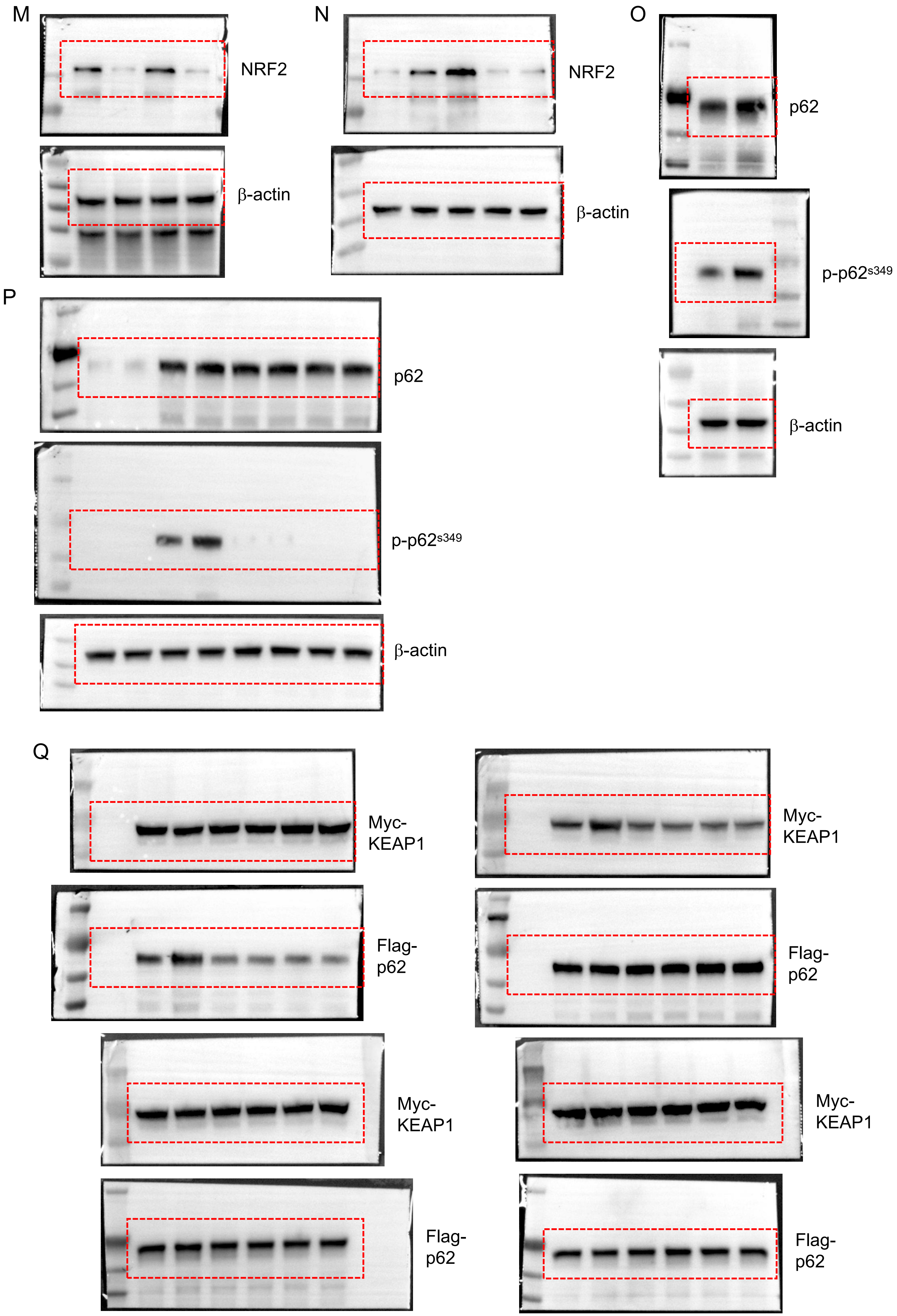

M
NRF2
β-actin
N
NRF2
β-actin
O
p62
p-p62s349
β-actin
P
p62
p-p62s349
β-actin
Q
Myc-
KEAP1
Flag-
p62
Myc-
KEAP1
Flag-
p62
Myc-
KEAP1
Flag-
p62
Myc-
KEAP1
Flag-
p62

Figure 6

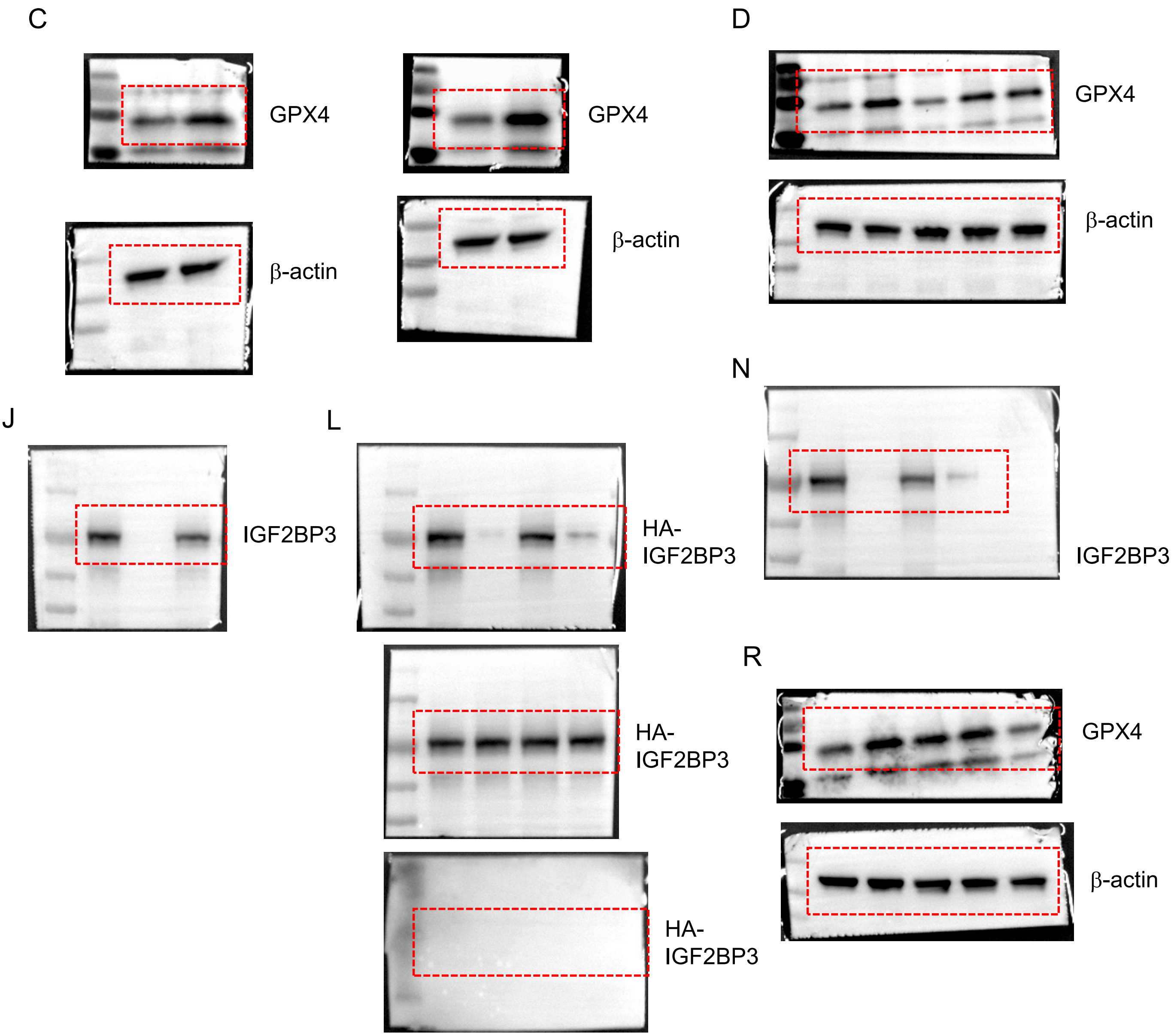

Supplementary Figure 8

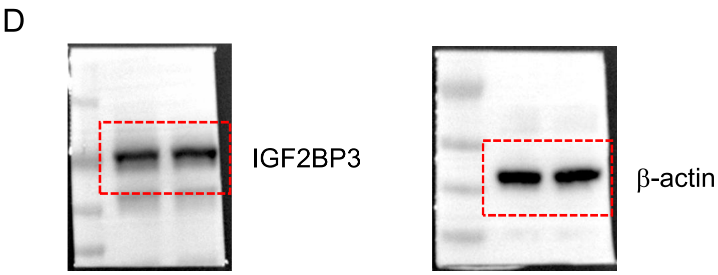

Supplement: Supplementary file 3 — Supporting Information [file ADVS-12-e01042-s001.pdf]
